# Emerging opportunities to treat drug-resistant breast cancer: Discovery of novel small-molecule inhibitors against different targets

**DOI:** 10.3389/fphar.2025.1578342

**Published:** 2025-08-29

**Authors:** Ruixian Chen, Zhilan Zhou, Xiangyue Meng, Yi Lei, Yuxi Wang, Yanyan Wang

**Affiliations:** ^1^ Department of Respiratory and Critical Care Medicine and Targeted Tracer Research and Development Laboratory and State Key Laboratory of Biotherapy and Cancer Center and Department of General Surgery and Breast Center/National Clinical Research Center for Geriatric & Nursing Key Laboratory of Sichuan Province, West China Hospital, Sichuan University, Chengdu, Sichuan, China; ^2^ General Practice Ward/International Medical Center Ward, General Practice Medical Center, West China Hospital, Sichuan University, Chengdu, Sichuan, China

**Keywords:** breast cancer, resistance, cell cycle checkpoint, drug design, structure-activity relationships

## Abstract

Breast cancer is now the most common malignancy among females. Numerous highly effective small-molecule inhibitors, such as tamoxifen and fulvestrant, have received clinical approval, significantly enhancing the therapeutic landscape of breast cancer. However, the emergence of drug resistance in some patients presents an unmet clinical need. Identifying resistance-related targets has been a key therapeutic strategy in combating drug-resistant tumors. Studies investigating the molecular characteristics and resistance mechanisms of breast cancer have unveiled novel targets, including cell cycle checkpoint molecules, breast cancer stem cell-related molecules, and anti-apoptotic proteins. Relevant small-molecule inhibitors have shown promising potential in overcoming drug-resistant breast cancer in both preclinical and clinical trials. In this review, we evaluate the resistance mechanisms of available targeted inhibitors for breast cancer and summarize innovative targeted strategies with the potential to overcome resistance, guided by clinical research.

## 1 Introduction

Breast cancer is the most prevalent malignant tumor among women and a leading cause of cancer-related mortality, posing a substantial threat to public health (Tao et al., 2015; Harbeck and Gnant, 2017). In recent years, small-molecule inhibitors characterized by both potent antitumor activity and favorable safety profiles have emerged as a cornerstone in the treatment of breast cancer, significantly reducing recurrence and mortality rates (Tryfonidis et al., 2016). Accordingly, this review focuses on targeted small-molecule inhibitors associated with breast cancer.

**FIGURE 1:**
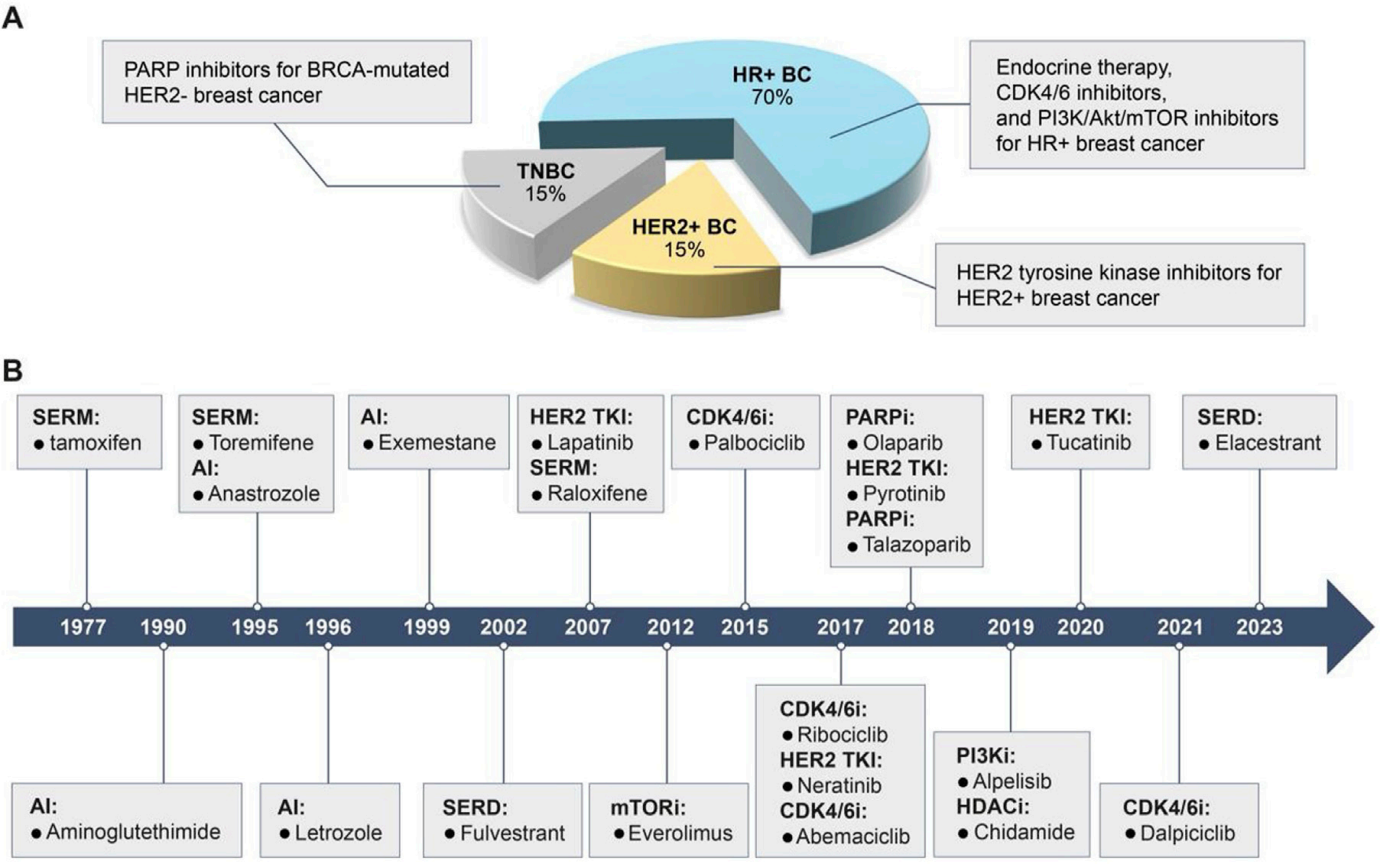
Breast cancer subtypes, characteristics, and clinically available target drugs. **(A)** Based on the expression of HR, HER2 and Ki67, breast cancer is classified as HR + breast cancer, HER2+ breast cancer and TNBC. HR + breast cancer is sensitive to endocrine therapy, CDK4/6 inhibitors and PI3K/Akt/mTOR inhibitors; HER2+ breast cancer is sensitive to HER2 TKIs; TNBC with BRCA mutations is sensitive to PARPi. **(B)** Timeline for the approval of clinically available small molecule inhibitors for breast cancer.

Breast cancer can be classified into several subtypes based on the expression of hormone receptors [HR, including estrogen receptor (ER) and progesterone receptor], human epidermal growth factor receptor 2 (HER2), and Ki67 (Figure 1A): HR-positive, HER2-positive, and triple-negative breast cancer (TNBC) (Waks and Winer, 2019). Despite advancements in therapeutic agents, a significant proportion of patients still experience tumor recurrence, metastasis, or disease progression due to the development of acquired resistance to initial treatments, ultimately resulting in mortality (Portman et al., 2019). This resistance arises from target gene mutations, activation of compensatory signaling pathways, and dysregulation of cell cycle checkpoints, apoptosis, or the tumor microenvironment (Lykkesfeldt et al., 2018; Zhou et al., 2020; Hanker et al., 2020). Once resistance emerges, patients are often shifted to alternative therapies targeting different molecular pathways. We have summarized the currently available targeted small-molecule inhibitors for various breast cancer subtypes and the salvage therapies employed following the failure of these inhibitors (Table 1). However, in the case of TNBC, with the exception of a minority of patients harboring breast cancer susceptibility gene 1/2 (BRCA1/2) mutations who are eligible for treatment with poly ADP-ribose polymerase (PARP) inhibitors, the majority continue to rely on conventional chemotherapy. Therefore, Table 1 includes only the inhibitors used in HR+ and HER2+ breast cancers (Waks and Winer, 2019).

**TABLE 1:** Clinical therapeutic landscape of small molecule inhibitors for breast cancer.

Tumor classification	Clinically available small molecule inhibitors	Rescue measures after treatment failure			
		Clinical status	Level I Recommendations	Level II Recommendations	Level III Recommendations
HER2+ breast cancer	HER2 targeted small molecule TKIs	Failure of HER2 TKI therapy	—	1. T-Dxd 2. trastuzumab, Pertuzumab + chemotherapy 3. T-DM1	Another category of TKIs + chemotherapy
HR + advanced breast cancer	endocrine therapy (include SERMs, SERDs and AI)	Failure of tamoxifen therapy	AI + CDK4/6i (Palbociclib, abemaciclib)	1. AI + HDAC inhibitor (Chidamide) 2. AI + CDK4/6i (Ribociclib) 3. AI + CDK4/6i (Dalpiciclib) 4. AI + mTOR inhibitor (Everolimus)	1. AI 2. SERD (Fulvestrant)
		Failure of non-steroidal AI therapy	SERD (Fulvestrant) + CDK4/6i (Palbociclib, abemaciclib, Dalpiciclib)	1. Steroidal AI + HDAC inhibitor (Chidamide) 2. SERD (Fulvestrant) + CDK4/6i (Ribociclib) 3. Steroidal AI + mTOR inhibitor (Everolimus)	1. SERD (Fulvestrant) 2. Steroidal AI 3. SERM (tamoxifen, Raloxifene) 4. progesterone
		Failure of steroidal AI therapy	SERD (Fulvestrant) + CDK4/6i (Palbociclib, abemaciclib, Dalpiciclib)	1. SERD (Fulvestrant) + CDK4/6i (Ribociclib) 2. SERD (Fulvestrant) + mTOR inhibitor (Everolimus) 3. Non-steroidal AI + CDK4/6i	1. SERD (Fulvestrant) 2. Non-steroidal AI 3. SERM (tamoxifen, Raloxifene) 4. progesterone
	CDK4/6 inhibitor	Failure of CDK4/6i therapy	—	1. Another CDK4/6i + endocrine therapy 2. Other targeted agents (such as everolimus, chidamide, and alpelisib) + endocrine therapy	1. progesterone 2. SERM (Toremifene)
	PI3K/Akt/mTOR inhibitor	—	—	—	—

Nevertheless, the limited efficacy of current salvage treatments underscores the urgent need for novel drugs and strategies to overcome therapeutic resistance (Hanker et al., 2020). Emerging targets—such as immune checkpoints, proteins associated with BCSCs, and apoptosis-related molecules—have shown promise in addressing resistance (Xi and Ma, 2020). Moreover, targeted small-molecule drugs continue to be a primary focus in the treatment of resistant tumors due to their structural flexibility, streamlined development process, potent antitumor activity, and reduced toxicity (Pisa and Kapoor, 2020). This review, therefore, provides a comprehensive overview of the mechanisms underlying resistance to small-molecule inhibitors in breast cancer and introduces novel therapeutic targets, primarily involving the cell cycle, BCSCs, DNA damage repair (DDR), and apoptotic pathways. It also outlines the developmental progress and clinical research advancements of small-molecule inhibitors targeting these mechanisms in breast cancer therapy.

## 2 Breast cancer treatment: small molecule drugs and mechanisms of drug resistance

The introduction of targeted small-molecule inhibitors has transformed the therapeutic landscape of breast cancer. Cyclin-dependent kinase 4/6 inhibitors (CDK4/6i) and phosphatidylinositol 3-kinase (PI3K)/protein kinase B (Akt)/mammalian target of rapamycin (mTOR) pathway inhibitors are utilized in HR + breast cancer, while HER2 tyrosine kinase inhibitors (TKIs) and PARP inhibitors (PARPi) are used to treat HER2+ and BRCA1/2-mutant breast cancers, respectively. These agents provide less toxic alternatives to traditional chemotherapy, making them more suitable for long-term administration (Waks and Winer, 2019).

However, the selective pressure exerted by these therapies on proliferating tumor cells inevitably leads to resistance (Pisa and Kapoor, 2020), which is mediated by diverse and complex mechanisms. This section presents an overview of the resistance mechanisms to endocrine therapy, HER2 TKIs, CDK4/6i, PARPi, and PI3K/Akt/mTOR inhibitors, and summarizes the current therapeutic strategies (Table 2; Figure 1B).

**TABLE 2:** Targeted small-molecule inhibitors applicable for breast cancer treatment.

Drug	Target	Structure	Activity	Breast cancer-related Indication	First approval status
Tamoxifen	ER (SERM)	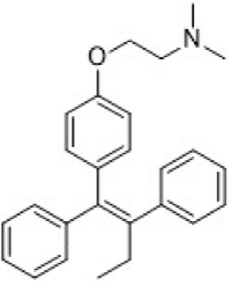	—	Recurrent or metastatic breast cancer	Approve by US FDA (12/1977)
Toremifene	ER (SERM)	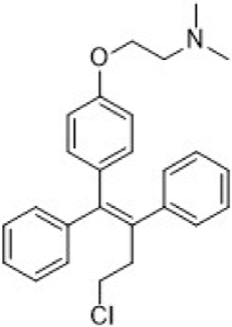	—	ER + metastatic breast cancer	Approve by Japan PMDA (03/1995)
Raloxifene	ER (SERM)	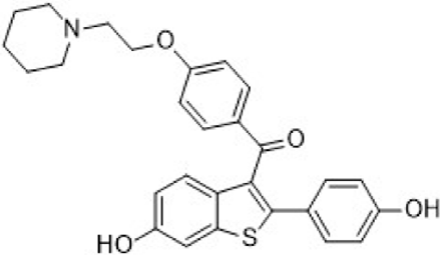	—	Breast cancer	Approve by US FDA (09/2007)
Fulvestrant	ER (SERD)	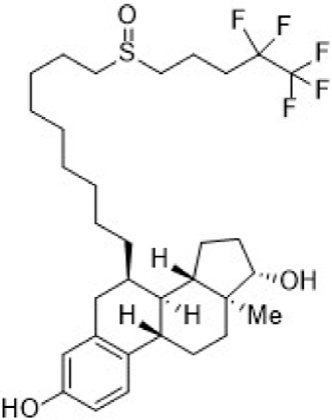	—	HR + metastatic breast cancer	Approve by US FDA (04/2002)
Elacestrant	ER (SERD)	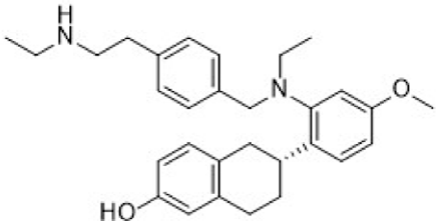	—	ER+/HER2-, ESR1-mutated advanced or metastatic breast cancer	Approve by US FDA (01/2023)
Aminoglut-ethimide	Aromata-se	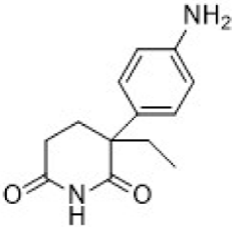	Aromatase IC_50_ = 10 μM	Breast cancer	Approve by China NMPA (01/1990)
Anastrozole	Aromata-se	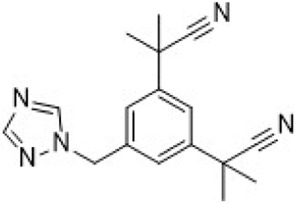	Aromatase IC_50_ = 14.6 nM	Advanced breast cancer in postmenopausal women	Approve by UK MHRA (08/1995)
Letrozole	Aromata-se	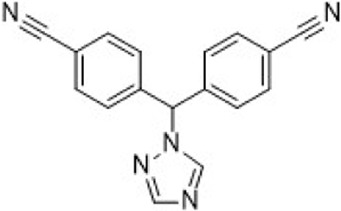	Aromatase IC_50_ = 11.5 nM	Advanced breast cancer	Approve by EU EMA (07/1996)
Exemestane	Aromata-se	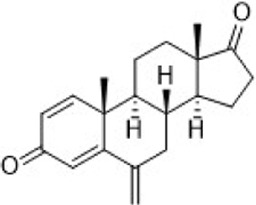	Aromatase IC_50_ = 30 nM	Breast cancer	Approve by US FDA (10/1999)
Lapatinib	HER2/EGFR	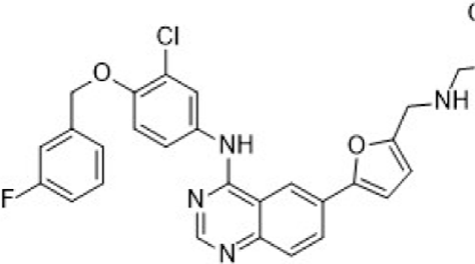	HER2 IC_50_ = 9.2 nM EGFR IC_50_ = 10.8 nM	Advanced or metastatic breast cancer	Approve by US FDA (03/2007)
Neratinib	HER2/EGFR/HER4	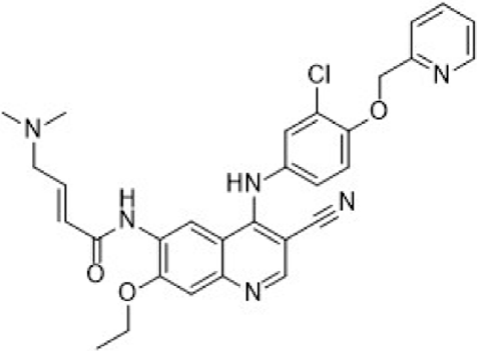	HER2 IC_50_ = 59 nM EGFR IC_50_ = 94 nM	HER2-overexpressed breast cancer	Approve by US FDA (07/2017)
Pyrotinib	HER2/EGFR	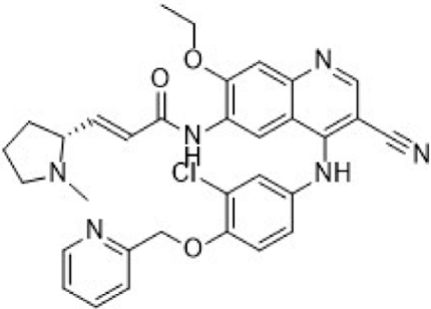	HER2 IC_50_ = 38 nM EGFR IC_50_ = 13 nM	Recurrent or metastatic breast cancer	Approve by China NMPA (08/2018)
Tucatinib	HER2	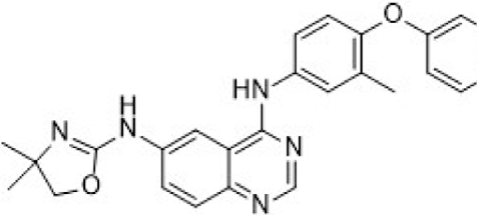	HER2 IC_50_ = 8 nM	Advanced unresectable or metastatic HER2+ breast cancer	Approve by US FDA (04/2020)
Palbociclib	CDK4/6	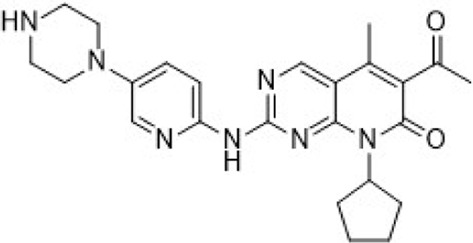	CDK4 IC_50_ = 11 nM CDK6 IC_50_ = 16 nM	ER+/HER2- advanced breast cancer	Approve by US FDA (02/2015)
Ribociclib	CDK4/6	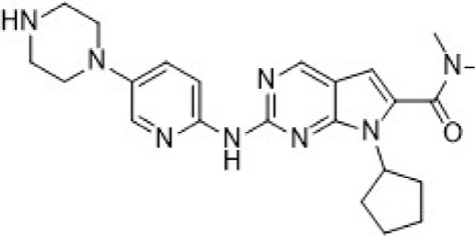	CDK4 IC_50_ = 10 nM CDK6 IC_50_ = 39 nM	HR+/HER2- advanced or metastatic breast cancer	Approve by US FDA (03/2017)
Abemacicl-ib	CDK4/6	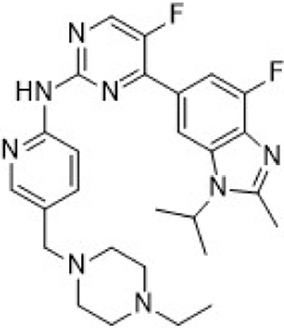	CDK4 IC_50_ = 2 nM CDK6 IC_50_ = 10 nM	HR+/HER2-advanced or metastatic breast cancer	Approve by US FDA (09/2017)
Dalpiciclib	CDK4/6	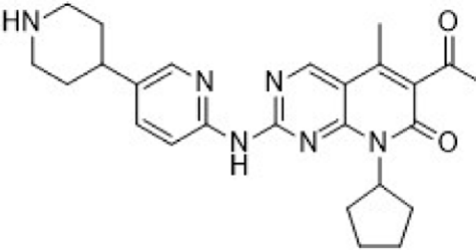	CDK4 IC_50_ = 12.4 nM CDK6 IC_50_ = 9.9 nM	HR+/HER2- Recurrent or metastatic breast cancer	Approve by China NMPA (12/2021)
Olaparib	PARP1/2	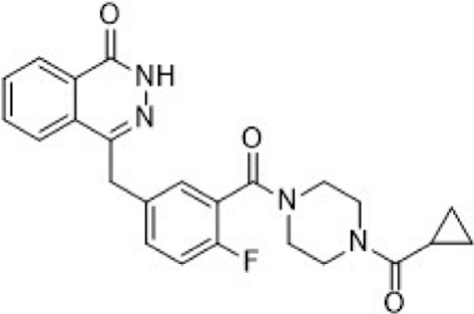	PARP1 IC_50_ = 5 nM PARP2 IC_50_ = 1 nM	Germline BRCA-mutated HER2- metastatic breast cancer	Approve by US FDA (01/2018)
Talazoparib	PARP1/2	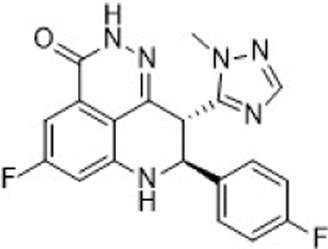	PARP1 IC_50_ = 0.58 nM	Germline BRCA-mutated HER2- locally advanced or metastatic breast cancer	Approve by US FDA (10/2018)
Alpelisib	PI3K	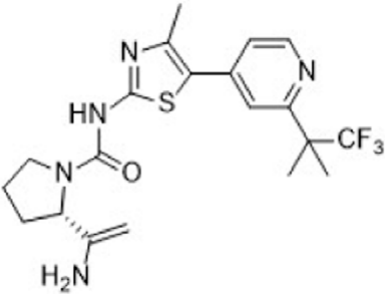	PI3Kα IC_50_ = 4.6 nM	HR+/HER2-, PIK3CA-mutated, advanced or metastatic breast cancer	Approve by US FDA (05/2019)
Everolimus	mTOR	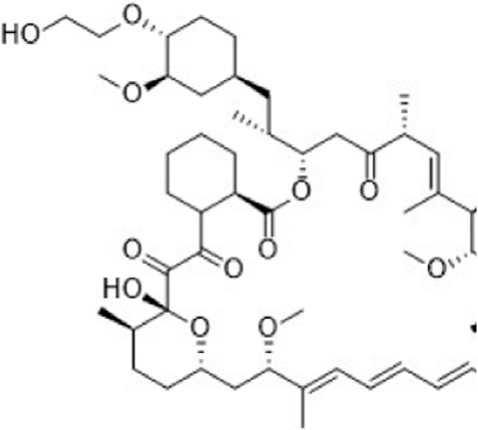	mTORC1 IC_50_:1.6–2.4 nM	HR-positive, HER2-negative breast cancer	Approve by US FDA (07/2012)
Chidamide	HDAC	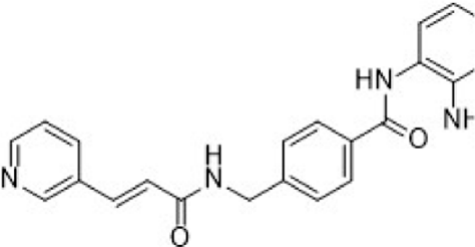	HDAC1/2/3 IC_50_ = 95/160/67 nM	HR+/HER2- breast cancer	Approve by US FDA (11/2019)

### 2.1 Endocrine therapy and mechanisms of drug resistance

Endocrine therapy—including selective estrogen receptor modulators (SERMs), selective estrogen receptor degraders (SERDs), and aromatase inhibitors (AIs)—primarily functions by modulating hormone levels in breast cancer patients, thereby suppressing tumor growth. Due to its relatively mild side-effect profile, endocrine therapy has become widely adopted in the treatment of HR + breast cancer. Nonetheless, prolonged use frequently results in the development of drug resistance, ultimately compromising treatment efficacy (Agg et al., 2019).

#### 2.1.1 SERM and SERD

The ER is a steroid HR and nuclear transcription factor, composed of two subtypes: ERα (encoded by the ESR1 gene) and ERβ (encoded by the ESR2 gene). These subtypes are widely distributed in various human tissues (Jameera Begam et al., 2017). Estrogens play a crucial role in many physiological activities in the human body, primarily through their interaction with ERs. When endogenous estrogens, particularly estradiol (E2), bind to cytoplasmic ERs, the receptors dimerize and translocate into the nucleus, guided by nuclear localization signals. The ER-E2 dimer complex then directly binds to the promoters of target genes or nearby estrogen response elements (EREs), recruiting various co-factors to activate or inhibit the transcription and expression of these genes (Cui et al., 2013). Extensive data has confirmed that the overactivation of endogenous estrogens is crucial in the initiation and development of breast cancer. Imbalances in the estrogen signaling pathway increase the risk of breast cancer, with ERα expression detected in approximately 75% of breast cancer cases (Brown, 2021). Therefore, targeting ER to block the estrogen signaling pathway represents a vital therapeutic strategy for ER-positive breast cancer patients. Currently, there are two main treatment approaches related to ERs.

SERMs compete with estrogen for binding to both ERα and ERβ, preventing the binding of coactivators. This inhibition suppresses the ER signaling pathway (Patel and Bihani, 2018; Brzozowski et al., 1997). SERMs exhibit varying estrogenic effects in different tissues. As of April 2023, three SERMs are globally approved: tamoxifen, toremifene, and raloxifene (Table 2).

SERDs interact with the ER and H12, causing conformational changes and structural disruption. This results in receptor ubiquitination and subsequent proteasomal degradation, ultimately abolishing the estrogen signaling pathway. As of September 2023, two SERDs are clinically approved globally: Fulvestrant and Elacestrant (Table 2).

#### 2.1.2 AIs

Inhibiting estrogen synthesis is another strategy for treating breast cancer (Waks and Winer, 2019). Aromatase (AR), a cytochrome P450 enzyme complex, converts androgens to estrogens in the body (androstenedione to estrogen, testosterone to estradiol) (Kharb et al., 2020; Yadav et al., 2015). It is responsible for the final step in estrogen biosynthesis. Thus, inhibiting AR can reduce estrogen levels in the body, counteracting breast cancer.

Based on their mechanism of action, AIs are classified into steroidal and nonsteroidal inhibitors. Steroidal inhibitors are either competitive inhibitors that resemble the substrate structure or suicide inhibitors. Nonsteroidal inhibitors primarily exert competitive inhibitory effects by forming coordination compounds with the heme of CYP-450 through a heteroatom (O, N, S) in their structure (Yadav et al., 2015). After years of development, several AIs have entered the market, including nonsteroidal AIs like Aminoglutethimide, Anastrozole, and Letrozole, and the steroidal AI Exemestane (Table 2).

In recent years, researchers have identified endocrine resistance as a significant challenge with the increasing use of endocrine therapy. This resistance is attributed to several factors: (Tao et al., 2015): changes in the quantity and affinity of ERs, and mutations in ER itself. For example, ER loss due to epigenetic changes can lead to resistance to tamoxifen and Fulvestrant (Fan et al., 2006). Additionally, single-point amino acid mutations in the ligand-binding domain (LBD) of ESR1 can cause constitutive activation of ER, enabling hormone-independent ER transcriptional activity and reducing the receptor’s sensitivity to drugs (Hanker et al., 2020; Katzenellenbogen et al., 2018); (Harbeck and Gnant, 2017) activation or mutation of pathways that interact with or compensate for ER (Razavi et al., 2018); (Tryfonidis et al., 2016) abnormal activation of growth factor receptor pathways or alternative survival pathways, such as overexpression of HER2 (Gutierrez et al., 2005), and activation of the Janus kinase (JAK)-signal transducers and activators of transcription 3 (STAT3) pathway, which can promote endocrine resistance by interacting with ER; (Waks and Winer, 2019); epigenetic modifications; cell cycle or mitotic molecules such as CDK2, CDK7, Monopolar spindle 1 (Mps1, also known as TTK), and Aurora kinase A (AURKA); dysregulation of apoptotic proteins; and the tumor microenvironment all contribute to endocrine resistance (Morotti et al., 2019; Brechbuhl et al., 2017; Finn et al., 2016). In fact, endocrine resistance is often driven by ligand-independent reactivation of ER (8).

### 2.2 HER2-targeted TKIs

HER2, a member of the epidermal growth factor receptor (EGFR) family (Yarden and Sliwkowski, 2001), does not form ligand-dependent homodimers for downstream signal activation under physiological conditions. It can only form heterodimers with activated family members, such as EGFR, or self-assemble into ligand-independent homodimers when overexpressed or activated (Bai et al., 2023; Lemmon et al., 2014). This leads to the phosphorylation of tyrosine kinase residues in the cytoplasmic domain, activating target genes and downstream signaling pathways (Bai et al., 2023; Hernández-Blanquisett et al., 2016). Abnormal expression of HER2 is associated with various tumors, including the occurrence, invasion, metastasis, proliferation, and poor prognosis of HER2-positive breast cancer (Liu et al., 2021). Targeted therapy against HER2 is one of the optimal treatment strategies for these malignant tumors (Perez et al., 2014; Slamon et al., 1989).

HER2-targeted small molecule TKIs, which can strongly penetrate the blood-brain barrier, inhibit the corresponding kinase and the phosphorylation of the tyrosine residues of its substrates, thereby disrupting downstream signal transduction (Huang et al., 2020). Currently, four TKIs are available for HER2+ breast cancer: (1) Lapatinib, which antagonizes EGFR and HER2; (2) Neratinib, a pan-HER irreversible TKI; (3) Pyrotinib, which antagonizes EGFR and HER2; (4) Tucatinib, which antagonizes HER2 (Table 2).

With the increasing application of HER2-TKI therapy, several potential resistance mechanisms to anti-HER2 treatment have been identified, including: (1) activating mutations in the HER2 gene; (2) tumor escape through the ER pathway (Giuliano et al., 2015); (3) abnormal activation of various alternative tyrosine kinases (Sun et al., 2020) or transcriptional dysregulation led by membrane receptors other than HER family; (4) alterations in downstream signaling pathways, such as mutations in the gene encoding phosphatidylinositol 3-kinase catalytic subunit (PIK3CA), which encodes the p110α subunit of PI3K (Berns et al., 2007), or excessive activation of the PI3K/Akt/mTOR pathway due to the downregulation of tumor suppressor genes (Nagata et al., 2004); (5) heterogeneity in HER2 expression within tumors (Filho et al., 2021); (6) the influence of the tumor microenvironment and cell cycle molecules. Ultimately, through pathway redundancy or stimulation of alternative survival pathways, these mechanisms lead to the reactivation of HER2 signaling pathway or its downstream cascades (Pernas and Tolaney, 2019).

### 2.3 CDK4/6i

CDKs play a crucial role in initiating the cell cycle and regulating transitions between various phases (Majumdar et al., 2021). Comprising 21 members, CDKs exhibit diverse functions: CDK1/2/4/6 govern cell cycle regulation, CDK7/8/9/11/12/13/19/20 are involved in transcriptional regulation, and CDK2/9/12 participate in DNA repair processes (Freeman-Cook KD. et al., 2021). In recent years, CDK4/6 have garnered considerable attention. Upon stimulation by mitotic signals, CDK4/6 bind to cyclin D, activating the complex and inducing phosphorylation of retinoblastoma (Rb). This leads to the dissociation of the transcriptional inhibitory complex Rb-E2F, releasing the transcription factor E2F. E2F activates related genes, mediating the cell’s entry into the S phase (Goel et al., 2018; Roberts et al., 2012). Normally, the activity of the CDK4/6-cyclin D complex is inhibited by various proteins, such as those from the inhibitor of CDK4 (INK4) family and CDK interaction protein/kinase inhibitor protein (CIP/KIP) (Sherr, 1996). However, in breast cancer, the activation of ER and other proliferative signals result in overactivation and amplification of CDK4/6 (Lange and Yee, 2011). Additionally, excessive cyclin D expression leads to sustained activation of the CDK4/6-cyclin D complex, ultimately resulting in uncontrolled cell proliferation (Braal et al., 2021). Therefore, drugs inhibiting CDK4/6 play a crucial role in the treatment of breast cancer.

CDK4/6i target the adenosine 5′-phosphate (ATP)-binding domain of proteins, inhibiting the formation of CDK4/6-cyclin D complexes and disrupting ATP binding. This interruption halts subsequent reaction cascades, suppressing DNA synthesis and tumor cell proliferation (Shi et al., 2022). Currently approved CDK4/6i are primarily used to treat HR+/HER2-breast cancer. They demonstrate partial reversibility in overcoming endocrine resistance. Examples include Palbociclib, Ribociclib, Abemaciclib, and Dalpiciclib (Table 2).

Despite the success of CDK4/6i, acquired resistance limits their widespread use. Changes leading to resistance can be categorized into two types: (Tao et al., 2015): cell cycle-specific resistance mechanisms, including the loss or mutation of the Rb protein (Spring et al., 2020), amplification of E2F, and the resulting increased phosphorylation of Rb by CDK2-cyclin E complexes (facilitating the entry of CDK2-mediated cells into the S phase) (Huang et al., 2022; Herrera-Abreu et al., 2016), amplification of CDK (promoting tumor progression through kinase-independent functions), overexpression of INK4 family members (inhibiting CDK4/6-cyclin D complexes, making tumor progression partially dependent on pathways outside CDK4/6) (Huang et al., 2022), and loss of expression of CIP/KIP family members (causing activation of the cyclin-CDK-Rb pathway) (Alvarez-Fernandez and Malumbres, 2020); (Harbeck and Gnant, 2017) cell cycle non-specific resistance mechanisms, including the activation of survival signaling pathways such as PI3K/Akt/mTOR and mitogen-activated protein kinase (MAPK) pathways (Kilker and Planas-Silva, 2006), loss or activity inhibition of ER activity (Yang et al., 2017), and activation of immune evasion pathways.

### 2.4 PARPi

BRCA1/2 mutations are common in tumors, leading to homologous recombination deficiency (HRD). As a result, cells rely on the error-prone non-homologous end-joining (NHEJ) pathway for DNA repair, causing chromosomal instability and impact the repair process of DNA double-strand breaks (DSB). This accumulation of DNA damage significantly increases cancer risk, including breast cancer (Hennessy et al., 2010; Zhao et al., 2020). Fortunately, tumors with BRCA1/2 mutations are sensitive to PARPi. The PARP enzyme family uses nicotinamide adenine dinucleotide (NAD+) as a substrate to synthesize poly (ADP-ribose), covalently adding it to target proteins through β-NAD+. This modification alters the function of target proteins and participates in various cellular processes, including DNA repair. Overall, PARP proteins are crucial in repairing DNA single strand breaks and maintaining genome stability (Altmeyer et al., 2009; De Vos et al., 2012).

The application of PARPi disrupts the catalytic activity of PARP, thereby preventing the necessary ADP-ribosylation required for subsequent DNA repair. This disruption simultaneously inhibits the repair of double-strand and single-strand DNA breaks in BRCA1/2 mutated cancer cells, leading to failed DNA repair and subsequent cell death, known as the “synthetic lethal effect” (30). Currently, the first-line PARPi for breast cancer are Olaparib and Talazoparib, both of which inhibit PARP1/2 (Table 2).

The efficacy of PARPi is limited to a small subset of patients with BRCA1/2 mutations. Most breast cancer patients with BRCA wild type exhibit intrinsic resistance to PARPi due to their intact DDR. Furthermore, the development of acquired resistance can result in PARPi treatment failure. Relevant mechanisms include: (1) Restoration of homologous recombination pathways, such as secondary mutations in genes like BRCA1/2, leading to functional recovery (Edwards et al., 2008) and an increase in the level of RAD51, a key component of the homologous recombination pathway (Weigelt et al., 2017); (2) Mutation of PARP or a reduction in intracellular PARP levels (Pettitt et al., 2013); (3) Restoration of protective mechanisms for replication forks independent of HR repair and DSB repair (Ray et al., 2016); (4) prolonged use of ATP-dependent drug efflux P-glycoprotein pump (P-gp) substrates like Olaparib leading to upregulation of P-gp, resulting in decreased intracellular concentrations of PARPi (Henneman et al., 2015); (5) Changes in DSB end resection, such as reduced dependence on PARP-dependent a-NHEJ associated with 53BP1 (Mirman et al., 2018); (6) Emergence of alternative factors in DSB repair, such as RAD52 (Sung and Klein, 2006) and DNA polymerase theta (Polθ) (Ceccaldi et al., 2015); (7) Enhancement of the DDR.

### 2.5 PI3K/Akt/mTOR inhibitors

The PI3K/Akt/mTOR signaling pathway plays a pivotal role within the complex network of cellular signaling in the human body. Activation of receptors such as receptor tyrosine kinases increases the activity of the PI3K complex, leading to the phosphorylation of PIP2 to generate PIP3. PIP3 then recruits Akt and phosphoinositide-dependent protein kinase 1 (PDK1) to the plasma membrane via its pleckstrin homology domain. Akt is activated through phosphorylation by PDK1 and mTOR complex 2 (mTORC2). Subsequently, downstream effector proteins such as mTOR complex 1 (mTORC1) are phosphorylated, thereby modulating cellular functions. Notably, mTORC1 regulates cellular processes, significantly contributing to bodily homeostasis (Bilanges et al., 2019). However, the PI3K/Akt/mTOR pathway is also highly susceptible to dysregulation in breast cancer. Dysregulation mechanisms include PIK3CA mutations (Saal et al., 2005) and inactivation of tumor suppressors like phosphatase and tensin homolog (PTEN) (Stemke-Hale et al., 2008), among others. Aberrant amplification of this pathway often leads to excessive cell proliferation, resistance to apoptosis, tumorigenesis, and progression (Verret et al., 2019).

Inhibitors of the PI3K/Akt/mTOR pathway include pan-PI3K inhibitors, selective PI3K subtype inhibitors, Akt inhibitors, mTOR inhibitors, PI3K/mTOR dual inhibitors, as well as other agents. These inhibitors may demonstrate efficacy as monotherapy in breast cancer with PIK3CA mutations, HER2 amplification, and tumor-suppressor PTEN defects. Two drugs have currently been approved for treating breast cancer patients: (1) Everolimus, which inhibits mTOR; and (2) Alpelisib, a selective inhibitor of PI3Kα (Table 2).

The emergence of inhibitors targeting the PI3K/Akt/mTOR pathway partially overcomes drug resistance induced by other medications. However, the inevitable acquired resistance poses a significant challenge. Resistance mechanisms include: (1) novel mutations in PIK3CA, PTEN (Razavi et al., 2020), and Akt, activating mutations in ESR1, as well as compensatory activation of the PI3K signaling pathway due to mutations; (2) Increased phosphorylation leading to Akt activation (Rong et al., 2020); (3) Upregulation of alternative survival pathways induced by drug exposure (Muranen et al., 2012); (4) Compensatory feedback loops activated by oncogenic genes such as MYC (Bihani et al., 2014).

Furthermore, the histone deacetylase (HDAC) inhibitor Chidamide was approved in China in 2019 for treating patients with locally advanced or metastatic HR+/HER2-breast cancer. However, due to the drug’s recent market introduction, discussions on resistance to HDAC inhibitors in breast cancer are currently deferred.

## 3 Emerging targeted therapeutic strategies to overcome drug-resistant breast cancer

**FIGURE 2:**
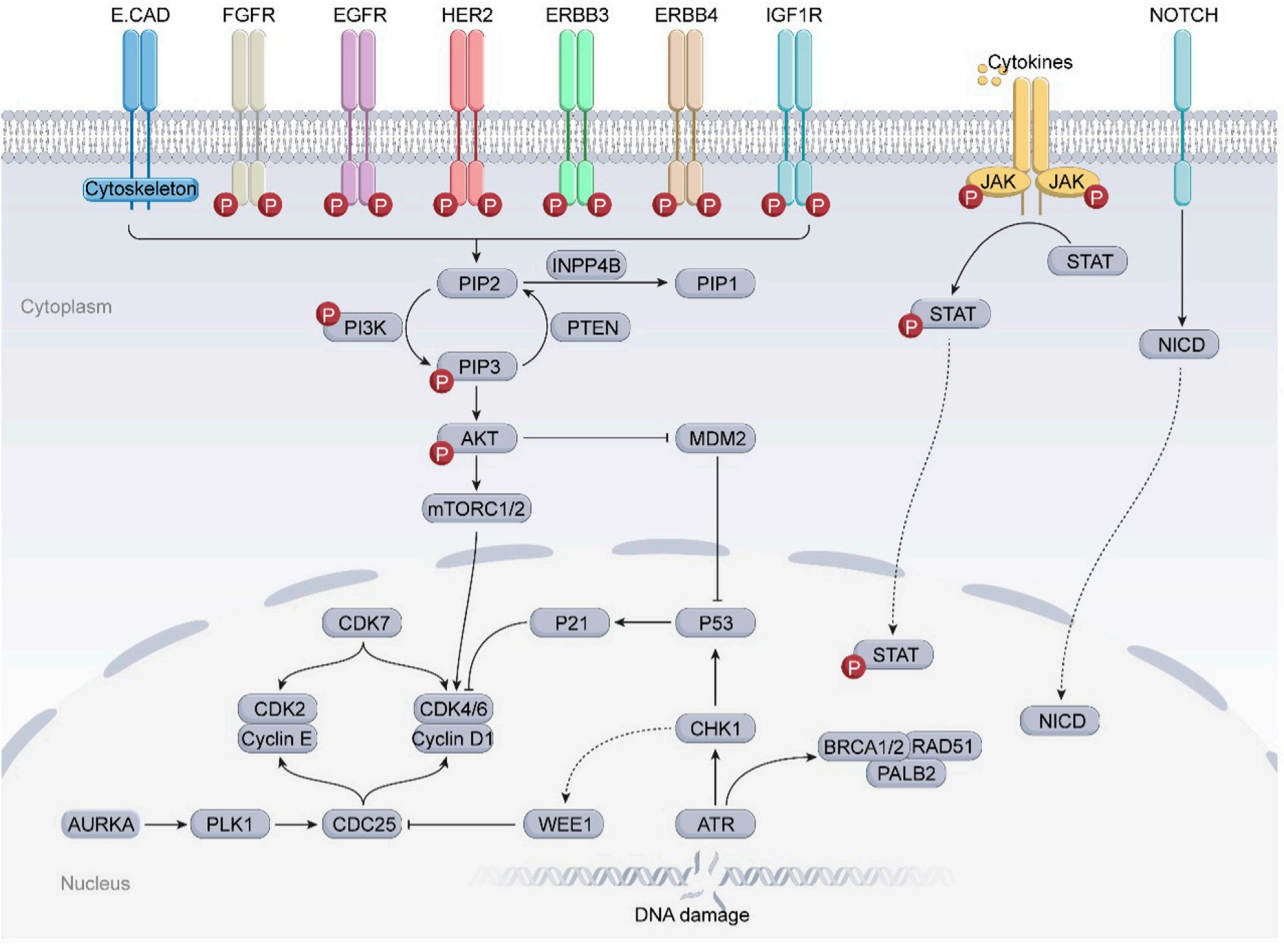
Several potential targets that may overcome drug resistance and relevant signaling pathways in breast cancer. The JAK-STAT3 and Notch signaling pathways, frequently found to be deregulated in breast cancer, are strongly associated with BCSCs and may significantly correlate with the resistance of breast cancer to clinically available small molecule inhibitors. In addition, the overexpression of cell cycle checkpoint components (such as CDKs, AURKA, ATR/CHK1/WEE1) and aberrant proteins (such as MDM2, RAD51) may substantially contribute to the progression of drug-resistant breast cancers. Further preclinical evidence supports the potential of these proteins to serve as novel targets for overcoming drug resistance in breast cancer.

Intricate tumor evolution, resistance mechanisms, and patient heterogeneity underscores the limited availability of effective drugs (Hanker et al., 2020). In this section, we take a clinical perspective, integrating the previously discussed drug resistance mechanisms in breast cancer, and investigate emerging targets to overcome resistance (Figure 2; Table 3).

**TABLE 3:** Novel targets and ongoing clinical trials in breast cancer.

Drug	Target	Phase	Indication	Interventions/Combination	NCT identifier	Status
PF-07104091	CDK2	II	Breast Cancer Solid Tumors	PF-07220060/fulvestrant/letrozole	NCT05262400	Recruiting
		I/II	Small Cell Lung Cancer Ovarian Cancer Breast Cancer	Palbociclib/fulvestrant/letrozole	NCT04553133	Recruiting
ARTS-021	CDK2	I/II	Solid Tumors	CDK 4/6 inhibitor/Fulvestrant/Letrozole/Carboplatin	NCT05867251	Not yet recruiting
BLU-222	CDK2	I/II	Advanced Solid Tumors HR + Breast Cancer CCNE1 Amplification HER2-negative Breast Cancer Ovarian Cancer	Carboplatin/Ribociclib/Fulvestrant	NCT05252416	Recruiting
INX-315	CDK2	I/II	Breast Cancer Breast Cancer Metastatic Hormone Receptor Positive Tumor Advanced Cancer	—	NCT05735080	Recruiting
PF-06873600	CDK2/4/6	II	HR + HER2- Metastatic Breast Cancer Ovarian Cancer Triple Negative Breast Cancer	Endocrine Therapy	NCT03519178	Active, not recruiting
SYH2043	CDK2/4/6	I	Advanced Malignant Tumors	—	NCT05728541	Not yet recruiting
PF-07224826	CDK2/4/6	I	Breast Cancer Ovarian Cancer Liposarcoma	Fulvestrant	NCT05905341	Not yet recruiting
RGT-419B	CDK2/4/6	I	Breast Cancer	Hormonal therapy	NCT05304962	Active, not recruiting
Q901	CDK7	I/II	Advanced Cancer Metastatic Cancer	Pembrolizumab	NCT05394103	Recruiting
Samuraciclib	CDK7	I/II	Inoperable, Locally Advanced or Metastatic, ER-positive Breast Cancer	Giredestrant/Abemaciclib/Ipatasertib/Inavolisib et al.	NCT04802759	Recruiting
SY-5609	CDK7	I	Advanced Solid Tumor Breast Cancer Small-cell Lung Cancer Pancreatic Cancer	Fulvestrant/Gemcitabine/Nab-paclitaxel	NCT04247126	Active, not recruiting
XL102	CDK7	I	Triple Negative Breast Cancer Hormone Receptor Positive Breast Cancer Neoplasm Malignant Epithelial Ovarian Cancer	Fulvestrant/Abiraterone/Prednisone	NCT04726332	Recruiting
TY-2699a	CDK7	I	Solid Tumor	—	NCT05866692	Not yet recruiting
CFI-402257	Mps1	I/II	ER/HER2- Advanced Breast Cancer	Fulvestrant	NCT05251714	Recruiting
		I/II	Advanced/Metastatic HER2- Breast Cancer	Paclitaxel	NCT03568422	Active, not recruiting
Alisertib	AURKA	II	Advanced, Endocrine-Resistant Breast Cancer	Fulvestrant/Laboratory Biomarker Analysis	NCT02860000	Active, not recruiting
		II	Locally recurrent or metastatic breast cancer	Paclitaxel	NCT02187991	Active, not recruiting
JAB-2485	AURKA	I/II	Solid Tumors ER + Breast Cancer Triple Negative Breast Cancer ARID1A Gene Mutation Small Cell Lung Cancer	—	NCT05490472	Recruiting
Ceralasertib	ATR	II	Triple Negative Breast Cancer Metastatic	Durvalumab/Nab-paclitaxel	NCT05582538	Recruiting
Berzosertib	ATR	I	Chemotherapy Resistant Triple Negative and Estrogen and/or Progesterone Receptor Positive, HER2 Negative Breast Cancer	Radiation therapy	NCT04052555	Recruiting
Prexasertib	CHK1/2	II	Chemotherapy-Pretreated Metastatic Triple Negative Breast Cancer	LY3023414	NCT04032080	Active, not recruiting
Adavosertib	WEE1	II	Metastatic Triple Negative Breast Cancer	Olaparib/Ceralasertib	NCT03330847	Active, not recruiting
Ruxolitinib	JAK1/2	II	Triple Negative Inflammatory Breast Cancer	Paclitaxel/Doxorubicin/Cyclophosphamide	NCT02876302	Active, not recruiting
TTI-101	STAT3	I/II	Breast Cancer	Palbociclib/Aromatase inhibitor	NCT05384119	Recruiting
AL101	γ-secretase	II	Notch-activated recurrent or metastatic Triple Negative Breast Cancer	—	NCT04461600	Active, not recruiting
CB-103	Notch Transcription Complex	II	Advanced Breast Cancer	Anastrozole/letrozole	NCT04714619	Active, not recruiting
X4P-001	CXCR4	I/II	Locally Advanced or Metastatic Triple Negative Breast Cancer	—	NCT05103917	Enrolling by invitation
Venetoclax	Bcl-2	I	ER and BCL-2 positive locally advanced or metastatic breast cancer	Palbociclib/Letrozole	NCT03900884	Recruiting
Navitoclax	Bcl-2 /Bcl-xL	I	High Grade Serous Carcinoma Triple Negative Breast Cancer	Olaparib	NCT05358639	Recruiting
APG-2575	Bcl-2	I/II	Solid Tumor ER+/HER2- metastatic breast cancer	Palbociclib	NCT04946864	Recruiting
TQB3909	Bcl-2	I/II	HR+/HER2- Advanced Breast Cancer	—	NCT05775575	Recruiting
Fadraciclib	CDK2/9	I/II	Solid Tumor HR+/HER2- metastatic breast cancer post-CDK4/6 inhibitor	—	NCT04983810	Recruiting
KB-0742	CDK9	I/II	Relapsed Solid Tumors Triple-negative breast cancer	—	NCT04718675	Recruiting
PRT2527	CDK9	I	Advanced Solid Tumors HR+/HER2- Breast Cancer	—	NCT05159518	Recruiting
JAB-3312	SHP2	I	Solid Tumors Non-small Cell Lung Cancer Breast Cancer	—	NCT04045496	Recruiting
		I	Solid Tumors Non-small Cell Lung Cancer Breast Cancer	—	NCT04121286	Recruiting
CYT-0851	RAD51	I/II	Relapsed/Refractory B-Cell Malignancies and Advanced Solid Tumors	Gemcitabine/Capecitabine/Rituximab/bendamustine	NCT03997968	Active, not recruiting
ART6043	Polθ	I/II	Advanced Solid Tumor HER2- locally advanced or metastatic breast cancer with a germline or somatic BRCA mutation	Olaparib/Talazoparib	NCT05898399	Recruiting
ART4215	Polθ	I/II	Advanced Cancer HER2- Breast cancer	Talazoparib/Niraparib	NCT04991480	Recruiting
Milademetan	MDM2	II	GATA3-mutant, ER+/HER- Advanced or Metastatic Breast Cancer	Fulvestrant	NCT05932667	Not yet recruiting
MPT0B640	Hsp90	Early Phase I	Advanced Solid Tumor Metastatic Solid Tumor	—	NCT04294875	Not yet recruiting

### 3.1 Targeting cell cycle checkpoint components

The cell cycle consists of the G1 (presynthetic growth), S (DNA synthesis), G2 (premitotic growth), and M (mitotic) phases. During the M phase, cells undergo mitosis. Cell cycle checkpoints—such as the G1/S checkpoint, intra-S checkpoint, G2/M checkpoint, spindle assembly checkpoint (SAC), and post-mitotic checkpoint—serve as critical regulatory mechanisms. When defects arise during DNA replication or mitosis, these checkpoints arrest cell cycle progression to allow for DNA repair. This arrest prevents premature or inappropriate transition to the next phase, which is essential for maintaining the integrity of DNA damage response (DDR) mechanisms (Malumbres and Barbacid, 2005). The DDR pathway integrates signals from both cell cycle checkpoints and DNA repair machinery into a cohesive network, thereby preventing the transmission of damaged DNA to daughter cells (Smith et al., 2020). In many cancers, including drug-resistant breast cancer, this regulatory precision is frequently disrupted due to altered expression or function of key checkpoint proteins and a general loss of checkpoint fidelity.

#### 3.1.1 CDK2 inhibitors

CDK2 is activated upon binding to cyclins E1, E2, A1, or A2, followed by phosphorylation by the cyclin-activating kinase (CAK)—comprising CDK7, cyclin H, and MAT1—and dephosphorylation of inhibitory sites by cell division cycle 25A (CDC25A) (Tadesse et al., 2020). Further activation cascade triggers the expression of several proteins essential for cell cycle progression, including cyclin A, the retinoblastoma protein (Rb), and enzymes involved in deoxyribonucleotide synthesis (Roskoski, 2016). The CDK2–cyclin complex plays a pivotal role in regulating the S phase and also phosphorylates proteins involved in DNA damage response, signal transduction, and apoptotic pathways, thereby integrating these processes into broader cellular functions (Tadesse et al., 2020; Chunder et al., 2012).

The dysregulation of CDK2 and its binding partners has been linked to poor clinical outcomes, reduced survival, and resistance to therapy (Hwang and Clurman, 2005). Oncogenic activation of CDK2–cyclin E1 impairs normal DNA replication, inducing replication stress and DNA damage, which leads to genomic instability and promotes malignant proliferation (Fagundes and Teixeira, 2021). In inflammatory breast cancer, CDK2 is critical for the maintenance and expansion of the breast cancer stem cell (BCSC) population (Opyrchal et al., 2014a). In hormone-dependent breast cancer, CDK2 phosphorylates estrogen and progesterone receptors, enhancing their transcriptional activity and thereby driving tumor progression (Rogatsky et al., 1999).

Given the central role of estrogen in cell cycle regulation, resistance to endocrine therapy is often associated with dysregulation of cell cycle-related genes and proteins, most of which converge on CDK2 activation. Therefore, CDK2 inhibition is a promising strategy to counteract these resistance pathways (Tadesse et al., 2020; Al-Qasem et al., 2022). Likewise, CDK2 plays a compensatory role in tumors that have acquired resistance to CDK4/6i, facilitating cell cycle progression despite upstream inhibition (Papadimitriou et al., 2022). Furthermore, CDK2 activity has also been implicated in partial resistance to HER2-targeted therapies in breast cancer (Smith et al., 2021; Decker et al., 2020).

**FIGURE 3:**
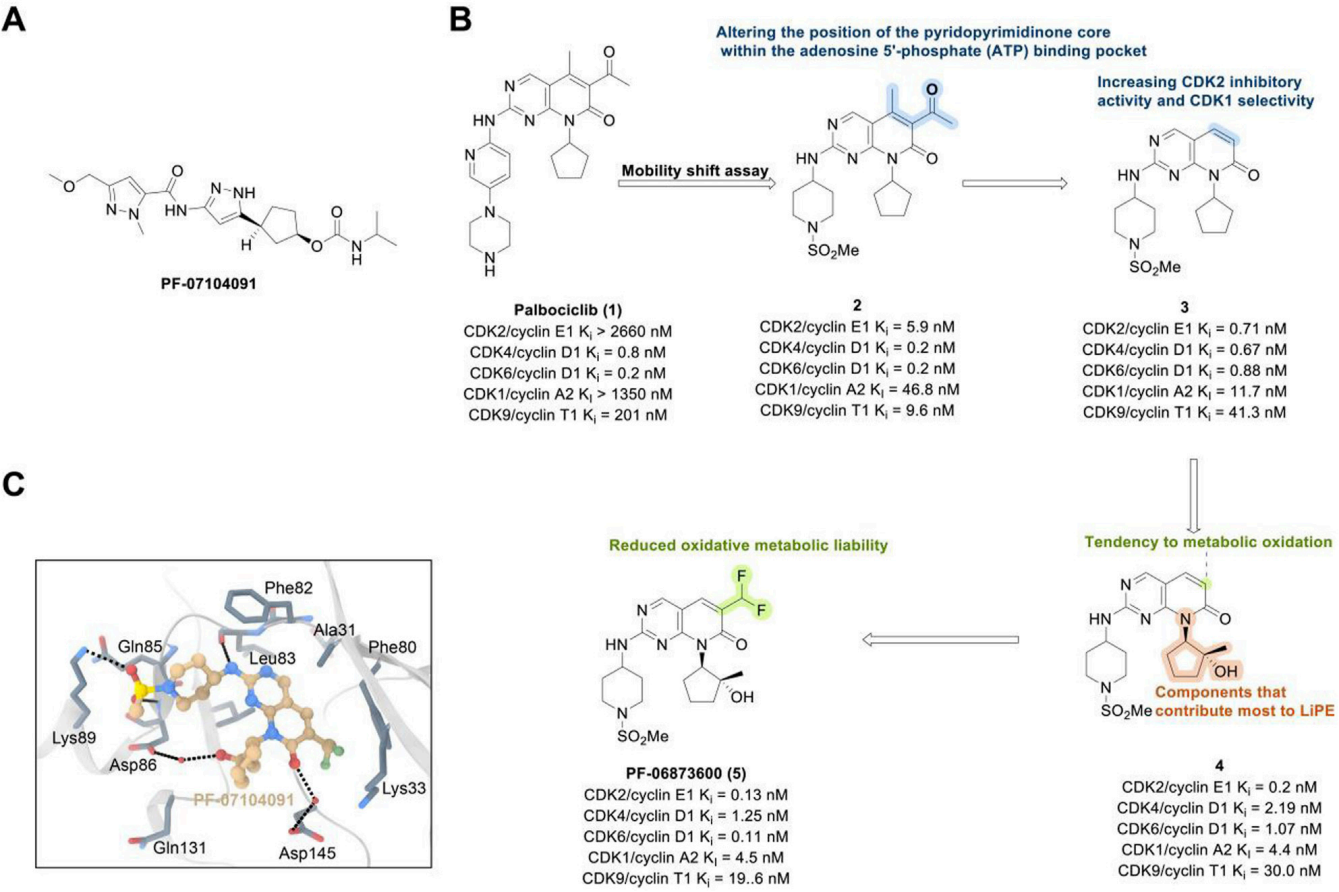
Chemical structures of inhibitors **one to five** and PF-07104091. **(A)** Chemical structure of PF-07104091. **(B)** Structure optimization process of compound **5**. **(C)** X-ray crystal structure of **five** in complex with CDK2 (PDB: 7KJS). Key residues, including Lys89 are highlighted in gray. Hydrogen bonds are shown in black.

The CDK2 inhibitor PF-07104091 is currently being evaluated in two clinical trials involving breast cancer patients (Figure 3A). These trials assess combinations with endocrine therapies such as Fulvestrant and Letrozole, alongside either the CDK4 inhibitor PF-07220060 (NCT05262400) or the CDK4/6i palbociclib (NCT04553133), and have shown encouraging early outcomes (Table 3) (Yap et al., 2023). The compound ARTS-021 is also under investigation in a trial that includes breast cancer patients with solid tumors (NCT05867251), as is BLU-222 in a similar study (NCT05252416). Additionally, INX-315 is currently in phase I/II trials for patients with recurrent or advanced/metastatic cancers, including HR+/HER2− breast cancer that has progressed following CDK4/6i therapy (NCT05735080).

Inhibitors targeting CDK2/4/6 present significant potential for further development (Table 3). For instance, PF-06873600 is under evaluation as a monotherapy and in combination with endocrine therapy for patients with HR+/HER2− metastatic breast cancer who have experienced progression after CDK4/6i treatment (NCT03519178). SYH2043 is being assessed in patients with advanced solid malignancies, including ER+/HER2− breast cancer (NCT05728541). In the second stage of a clinical trial, PF-07224826 will be combined with Fulvestrant for HR+/HER2− advanced or metastatic breast cancer patients who have previously received CDK4/6i or endocrine therapy (NCT05905341). A parallel trial is also being conducted for RGT-419B (NCT05304962). The continuous advancement of these investigational drugs reinforces the therapeutic relevance of CDK2 and propels the development of next-generation targeted small-molecule therapies for resistant breast cancer.

PF-06873600 is an effective inhibitor targeting CDK2/4/6 (41). Developers hypothesized that inhibiting CDK1 might lead to toxicity, making the development of compounds that effectively and selectively inhibit CDK2/4/6 while sparing CDK1 an attractive strategy. PF-06873600 is a pyridopyrimidinone-derived CDK2/4/6 inhibitor rationally designed from the palbociclib scaffold. Structure-based optimization—specifically the removal of the 5-methyl and 6-acetyl groups and the incorporation of a (1R,2R)-2-hydroxy-2-methylcyclopentyl moiety and a 6-difluoromethyl substituent—resulted in markedly enhanced CDK2 potency and kinase selectivity (Freeman-Cook KD. et al., 2021). The optimized compound demonstrated subnanomolar inhibition of CDK2 and over 30-fold selectivity against CDK1 and CDK9, along with favorable absorption, distribution, metabolism, and excretion properties. *In vitro*, PF-06873600 potently suppressed RB1 phosphorylation and inhibited cancer cell proliferation, while *in vivo* it achieved robust tumor regression, including approximately 90% reduction in OVCAR3 ovarian tumor xenografts at a single 50 mg/kg oral dose (Freeman-Cook K. et al., 2021). In diverse murine xenograft models driven by Myc or cyclin E amplification, including breast and ovarian cancers, PF-06873600 induced pronounced tumor growth inhibition. Importantly, it promoted tumor cell cycle arrest without impairing anti-tumor immune responses in immunocompetent syngeneic models (Freeman-Cook K. et al., 2021). Based on its potent preclinical efficacy, PF-06873600 progressed to a first-in-human Phase 1/2a clinical trial in patients with advanced solid tumors, including HR+/HER2-breast cancer, TNBC, and ovarian cancer. In endocrine-resistant HR + breast cancer, combination therapy with Fulvestrant yielded encouraging activity: partial responses were observed in both CDK4/6i-pretreated (objective response rate: 6.7%) and CDK4/6-naïve cohorts (22.7%), highlighting its potential to overcome CDK4/6 resistance (Yap et al., 2025). (Figures 3B,C).

RGT-419B is a third-generation CDK inhibitor featuring an optimized kinase activity spectrum. Developed through the Computer Accelerated Rational Design technology platform, it potently inhibits CDK2/4/6 while demonstrating favorable selectivity over other kinases, such as CDK9 and GSK3β. In palbociclib-resistant ER + breast cancer cell lines, RGT-419B exhibits stronger activity than abemaciclib in in vivo models. Furthermore, it shows superior anti-proliferative effects in ER + breast cancer cells with cyclin E1 overexpression. *In vivo*, RGT-419B sustains tumor growth inhibition (TGI) more effectively than abemaciclib in heterotransplanted ER + breast cancer models, underscoring its potential as a novel therapeutic option for patients with CDK4/6i-resistant or refractory ER + breast cancer (Xie et al., 2021). Currently, RGT-419B is undergoing Phase I clinical trials (NCT05304962), with encouraging preliminary results.

#### 3.1.2 CDK7 inhibitors

CDK7 plays a dual regulatory role in both cell cycle progression and transcriptional control. The CAK complex catalyzes the phosphorylation of the T-loop or threonine residues in the activation segment of CDK1/2/4/6, thereby facilitating their activation and indirectly participating in all phases of the cell cycle (Teng et al., 2019). In addition, the CAK complex is an essential component of the highly conserved transcription factor IIH (TFIIH) complex. TFIIH phosphorylates the C-terminal domain (CTD) of the large subunit of RNA polymerase II (RNA Pol II), promoting promoter clearance and initiating transcription (Rimel and Taatjes, 2018; Li et al., 2022). CDK7-mediated phosphorylation also regulates the activity of various transcription factors, including p53 and ER, thereby influencing the expression of downstream target genes (Chipumuro et al., 2014).

Aberrant CDK7 kinase activity has been observed in various tumors and is associated with aggressive clinicopathological features, including enhanced tumor progression and invasiveness (Diab et al., 2020). This dysregulation is particularly prominent in ER + breast cancer, where overexpression of all CAK complex components is frequently detected. CAK-mediated activation of ER drives tumor progression and may enhance the sensitivity of ER + breast cancer to CDK7 inhibition. Moreover, tumor cells rely more heavily than normal cells on transcription driven by super-enhancers activated by specific oncogenic drivers. This transcriptional dependency creates a therapeutic window: CDK7 inhibition reduces CTD phosphorylation, disrupts transcription elongation, induces transcriptional pausing, and ultimately leads to cell cycle arrest (Riedl and Egly, 2018).

**FIGURE 4:**
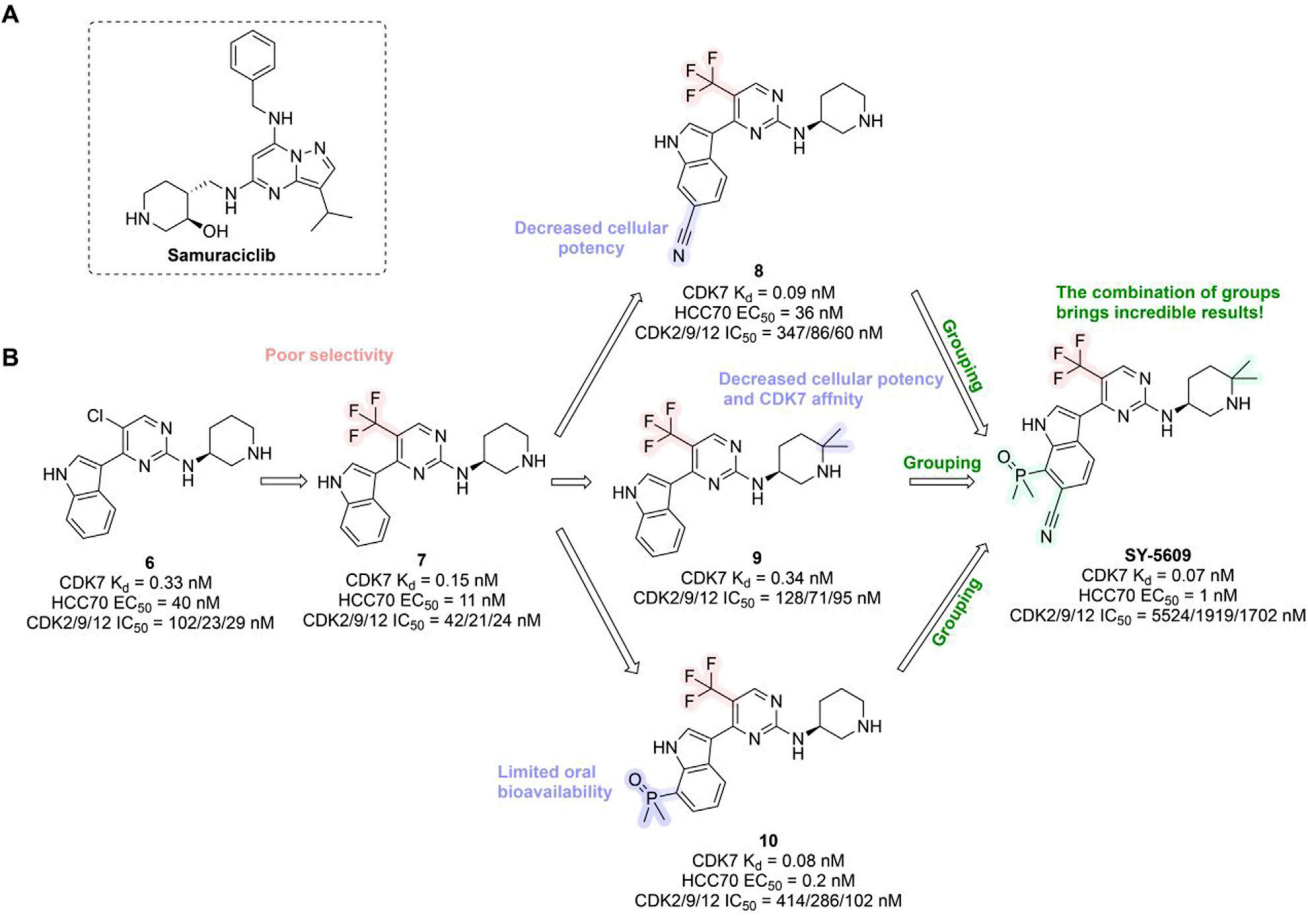
Chemical structures of inhibitors **6**-**10,** SY-5609 and Samuraciclib. **(A)** Chemical structure of Samuraciclib. **(B)** Structure optimization process of SY-5609.

In preclinical settings, CDK7 inhibitors have demonstrated potential in overcoming resistance in breast cancer. Given the essential role of CDK7 in both wild-type and mutated forms of ER-positive breast cancer and its association with reduced responsiveness to tamoxifen, CDK7 inhibitors may help counteract resistance due to ER mutations (Harrod et al., 2017; Jeselsohn et al., 2018). Additionally, these inhibitors can enhance tamoxifen sensitivity in ER-positive breast cancer by suppressing cancer stemness (Attia et al., 2022). Furthermore, CDK7 inhibitors can impede the activation of genes regulated by multiple HER2 inhibitor-resistant kinases, offering an alternative therapeutic approach to reverse acquired resistance in HER2-positive breast cancer (Sun et al., 2020). Reprogramming of tumor cells towards kinases such as EGFR, CDK2, and CDK7 has been implicated in resistance to the CDK4/6i palbociclib in ER-positive breast cancer (Pancholi et al., 2020). The heterogeneity within molecular subtypes—particularly between Luminal A and Luminal B—may also have a substantial impact on therapeutic response. Luminal B tumors demonstrate elevated expression of proliferation-related genes (e.g., CCNB1, MKI67, MYBL2), reduced estrogen/progesterone receptor signaling, and a higher frequency of genomic alterations such as TP53 mutations and HER2 co-amplification, contributing to endocrine resistance (Tran and Bedard, 2011). Cohort data from METABRIC and PAM50 further reveal that CDK7 and its partners (Cyclin H, MAT1) are significantly upregulated in Luminal B tumors (Patel et al., 2016). These features suggest that Luminal B tumors may be more sensitive to CDK7 inhibition and highlight the potential of subtype-guided targeted strategies to overcome resistance. Therefore, the use of CDK7 inhibitors in breast cancer may delay the onset of resistance, leading to more sustained responses. A preclinical study demonstrated that CDK7 inhibitors, such as THZ1, significantly suppressed the growth of resistant HER2-positive breast cancer cells (SKBR3, BT474, MDAMB453, HCC1954, HCC1569 and MDAMB361, average IC_50_ = 60 nM), and reduced phosphorylation of RNA polymerase II, thereby impairing cell transcriptional activity and enhancing apoptosis (Sun et al., 2020). Similarly, CDK7 inhibitors exhibit strong synergy with the EGFR inhibitor erlotinib in TNBC cell lines, markedly improving therapeutic efficacy (McDermott et al., 2020). These findings suggest that CDK7 inhibition may overcome genotype-dependent resistance by simultaneously disrupting multiple oncogenic pathways through suppression of proliferative signaling. Currently, selective CDK7 inhibitors such as Q901, TY-2699a, XL-102, and SY-5609, are being explored as monotherapies or in combination with other drugs for treating advanced solid tumors, including breast cancer (Table 3). In an ongoing Phase Ib/II study (NCT04802759) involving breast cancer patients at locally advanced or metastatic stages who are ineligible for surgery and have previously received CDK4/6i or standard anti-HER2 therapy, the CDK7 inhibitor Samuraciclib (also known as ICEC-0942, CT-7001) is being investigated (Figure 4A).

During the development of the clinical CDK7 inhibitor SY-5609, early leads such as compound **6**—a 2-amino-4-indolylpyrimidine derivative—demonstrated potent CDK7 activity (K_d_ = 0.33 nM) but limited selectivity toward CDK2/9/12. Subsequent analogs (e.g., compound **7**) improved CDK7 potency but failed to achieve sufficient kinase selectivity. Further modifications on the indole ring, including a six-position nitrile to engage the P-loop and a sulfone isostere to enhance hydrogen bonding, improved stability but did not fully optimize the balance between potency, selectivity, and pharmacokinetic (PK) properties. Ultimately, the incorporation of a phosphine oxide group and a nitrile moiety, along with a gem-dimethyl piperidine to increase lipophilicity, led to the discovery of SY-5609 (Figure 4B) (Marineau et al., 2022). SY-5609 was found to be effective (CDK7 K_d_ = 0.07 nM, HCC70 EC_50_ = 1 nM), highly selective (CDK2/9/12 IC_50_ = 5524/1919/1702 nM), and stable in mouse liver microsomes, with significant oral bioavailability. It inhibited CDK7 in the CAK and TFIIH complexes, leading to reduced transcription in tumor cells, G2/M cell cycle arrest, downregulation of key oncogenes like c-MYC, and ultimately inducing apoptosis in cancer cells. At a dose of 2 mg/kg, SY-5609 induced tumor regression in a TNBC xenograft model, demonstrating potent anti-tumor activity. Clinically, SY-5609 is undergoing evaluation in early-phase trials (e.g., NCT04247126) for treatment-refractory solid tumors, including advanced HR + breast cancer following CDK4/6i failure, pancreatic ductal adenocarcinoma, and biliary tract malignancies. While objective responses have been infrequent, a meaningful proportion of patients achieved disease control. Notably, in heavily pretreated metastatic HR+/HER2-breast cancer, the combination of SY-5609 and Fulvestrant achieved disease stabilization in approximately 42% of evaluable patients, including those with prior Fulvestrant exposure and liver metastases (Juric et al., 2023).

#### 3.1.3 Mps1 inhibitors

Mps1 is a dual-specificity protein kinase (Winey et al., 1991). As a core component of the spindle assembly checkpoint (SAC)—a critical surveillance mechanism that ensures proper cell proliferation and division—Mps1 activity is essential for the formation of the mitotic checkpoint complex and activation of the SAC((Wang et al., 2019). By monitoring the bipolar attachment and tension of all chromosomes to spindle microtubules, Mps1 regulates the mitotic spindle checkpoint, thereby ensuring the accurate segregation of sister chromatids at kinetochores (Foijer et al., 2014). In addition to its role in mitosis, Mps1 is also involved in centrosome duplication, DNA damage checkpoint response, ciliogenesis, cellular transformation, organ development, and other biological processes (Liu and Winey, 2012).

Elevated Mps1 expression has been observed in various human tumors, including breast cancer, and serves as a significant prognostic marker (Dominguez-Brauer et al., 2015; Daniel et al., 2011). Inhibition of Mps1 disrupts SAC function, resulting in mitotic abnormalities in breast cancer cells, reducing their viability, inducing apoptosis, and ultimately decreasing survival (Győrffy et al., 2014). Notably, Mps1 inhibition has shown potential in overcoming resistance to CDK4/6i in ER + breast cancer. Research by Isabel Soria-Bretones et al. (2024) identified a subset of CDK4/6i-resistant tumors characterized by increased mitotic defects and micronuclei formation. Treatment with the Mps1 inhibitor CFI-402257 exacerbates genomic instability in these cells, producing strong anti-proliferative and cytotoxic effects. In RB1-deficient breast cancer models, CFI-402257 induces premature chromosome separation and excessive mitotic segregation, resulting in elevated DNA damage and enhanced genomic instability. In summary, Mps1 represents a promising therapeutic target for anticancer treatment. Currently, CFI-402257 is under clinical investigation in breast cancer (Table 3), in combination with Fulvestrant, as part of a trial evaluating its efficacy in patients with advanced ER+/HER2− breast cancer who have previously received CDK4/6i and endocrine therapy (NCT05251714).

**FIGURE 5:**
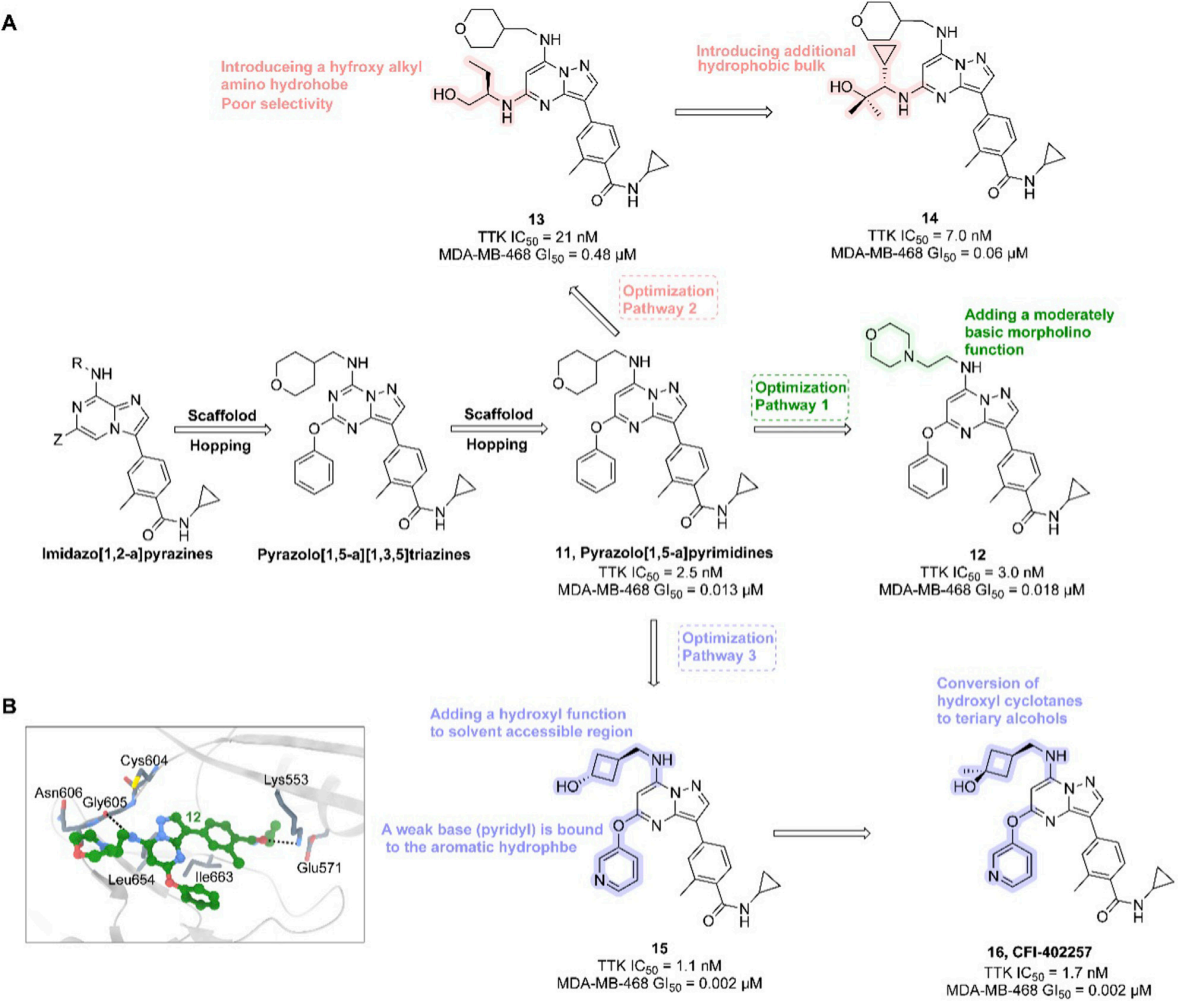
Chemical structures of inhibitors **11**-**16**. **(A)** Structure optimization process of compound **16**. **(B)** X-ray crystal structure of **16** in complex with Mps1 (PDB: 4ZEG). Key residues, including Lys553 are highlighted in gray. Hydrogen bonds are shown in black.

In the development of CFI-402257, researchers employed scaffold hopping from imidazo [1,2-a]pyrazines to pyrazolo [1,5-a]pyrimidines to improve Mps1 inhibitory potency and PK properties. The initial lead compound **11** (TTK IC_50_ = 2.5 nM, MDA-MB-468 GI_50_ = 0.013 μM) demonstrated potent activity but poor oral bioavailability. Optimization strategies included introducing polar, basic, solubilizing substituents (e.g., morpholino groups) into solvent-exposed regions, yielding compound **12** (TTK IC_50_ = 3.0 nM, MDA-MB-468 GI_50_ = 0.018 μM), which retained enzymatic potency but exhibited diminished antiproliferative efficacy. Crystal structural analysis confirmed tolerance of polar groups, prompting further modifications with hydroxyalkyl and hydrophobic moieties. These efforts produced compound **14** (TTK IC_50_ = 7.0 nM, MDA-MB-468 GI_50_ = 0.06 μM) with improved cellular activity. Subsequent refinement, including hydroxyl substitutions and coupling weakly basic pyridyl groups to aromatic hydrophobes, led to compound **16** (CFI-402257)—a tertiary alcohol analog of a cyclobutane derivative—with optimal potency (TTK IC_50_ = 1.7 nM, MDA-MB-468 GI_50_ = 0.002 μM), exquisite selectivity, and PK profile (Figure 5). CFI-402257 emerged as the most promising candidate and is currently in clinical evaluation (Liu et al., 2016). A first-in-human Phase I trial (NCT02792465) demonstrated that CFI-402257, either as monotherapy or in combination with Fulvestrant, exhibited a manageable safety profile in patients with solid tumors, including HR+/HER2– breast cancer. Investigator-assessed partial responses occurred in 8% of patients, and disease control was achieved in over 50%. Among HR+/HER2– patients previously treated with CDK4/6i and AI (n = 25), four partial responses were observed, with a median response duration of 223 days (Call et al., 2024). CFI-402257 has been granted Fast Track designation by the U.S. FDA and is currently undergoing dose confirmation and expansion studies in Phase I/II clinical trials.

#### 3.1.4 AURKA inhibitors

AURKA plays a vital role in orchestrating key events during mitosis (Yan et al., 2016). Prior to the onset of the M phase, AURKA associates with Bora, facilitating the phosphorylation and activation of polo-like kinase 1 (PLK1). PLK1, in turn, activates the CDK1–cyclin B complex by promoting the degradation of the CDK-inhibitory kinase WEE1 and the activation of the CDK-activating phosphatase CDC25C (Seki et al., 2008). AURKA also activates another CDK regulator, the phosphatase CDC25B, thereby further promoting the G2/M phase transition (Dutertre et al., 2004).

Overexpression or gene amplification of AURKA has been widely reported across various human cancers (Siggelkow et al., 2012). This dysregulation promotes cell cycle progression by removing inhibitory signals and enhancing the activity of cell cycle promoters. Additionally, AURKA modulates survival signaling and apoptotic regulators to support tumor cell survival and inhibit apoptosis. Its overexpression disrupts cell cycle checkpoints, induces aneuploidy and genomic instability, and plays a role in tumor invasion and metastasis by activating multiple oncogenic signaling pathways. Moreover, AURKA is centrally involved in maintaining tumor stem cell-like properties (Yan et al., 2016; Damodaran et al., 2017; Du et al., 2021; Pradhan et al., 2021). AURKA inhibitors impair mitotic spindle assembly, restrict cell cycle progression, and suppress AURKA activity, thereby inhibiting the proliferation, migration, and invasion of cancer cells (Pradhan et al., 2021).

AURKA inhibitors represent a potentially transformative strategy for overcoming drug resistance in breast cancer. AURKA overexpression has been shown to interact directly with ERα both *in vivo* and *in vitro*, promoting phosphorylation at Ser167 and Ser305 and enhancing ERα transactivation (Zheng et al., 2014). This phosphorylation can be further modulated by other molecules, increasing ERα DNA binding and transcriptional activity (Gao et al., 2018), thus inducing endocrine resistance in breast cancer and potentially serving as a biomarker of early resistance in ER + subtypes (Lykkesfeldt et al., 2018). Additionally, aberrant AURKA activity can downregulate ERα expression through alternative pathways, further contributing to endocrine resistance (Opyrchal et al., 2014b). AURKA amplification has also been implicated in resistance to CDK4/6i in ER+/HER2− breast cancer (Wander et al., 2020). In Rb1-deficient tumors—including CDK4/6i-resistant breast cancer—upregulation of mitosis-associated E2F target genes provides a therapeutic opportunity for AURKA inhibitors to trigger replication stress via synthetic lethality, leading to tumor cell death (Gong et al., 2019). AURKA may also attenuate the efficacy of PI3K-pathway inhibitors in breast cancer (Donnella et al., 2018). Thus, AURKA inhibitors hold great potential in breast cancer therapy (Table 3). Currently, the selective AURKA inhibitor alisertib is being investigated in clinical trials, either as monotherapy or in combination with fulvestrant, for patients with advanced endocrine-resistant breast cancer (NCT02860000). Another trial is evaluating alisertib in combination with paclitaxel for patients with HR+/HER2− or triple-negative locally recurrent or metastatic breast cancer (NCT02187991). In parallel, JAB-2485, another AURKA inhibitor, is under clinical evaluation in patients with solid tumors, including breast cancer (NCT05490472).

**FIGURE 6:**
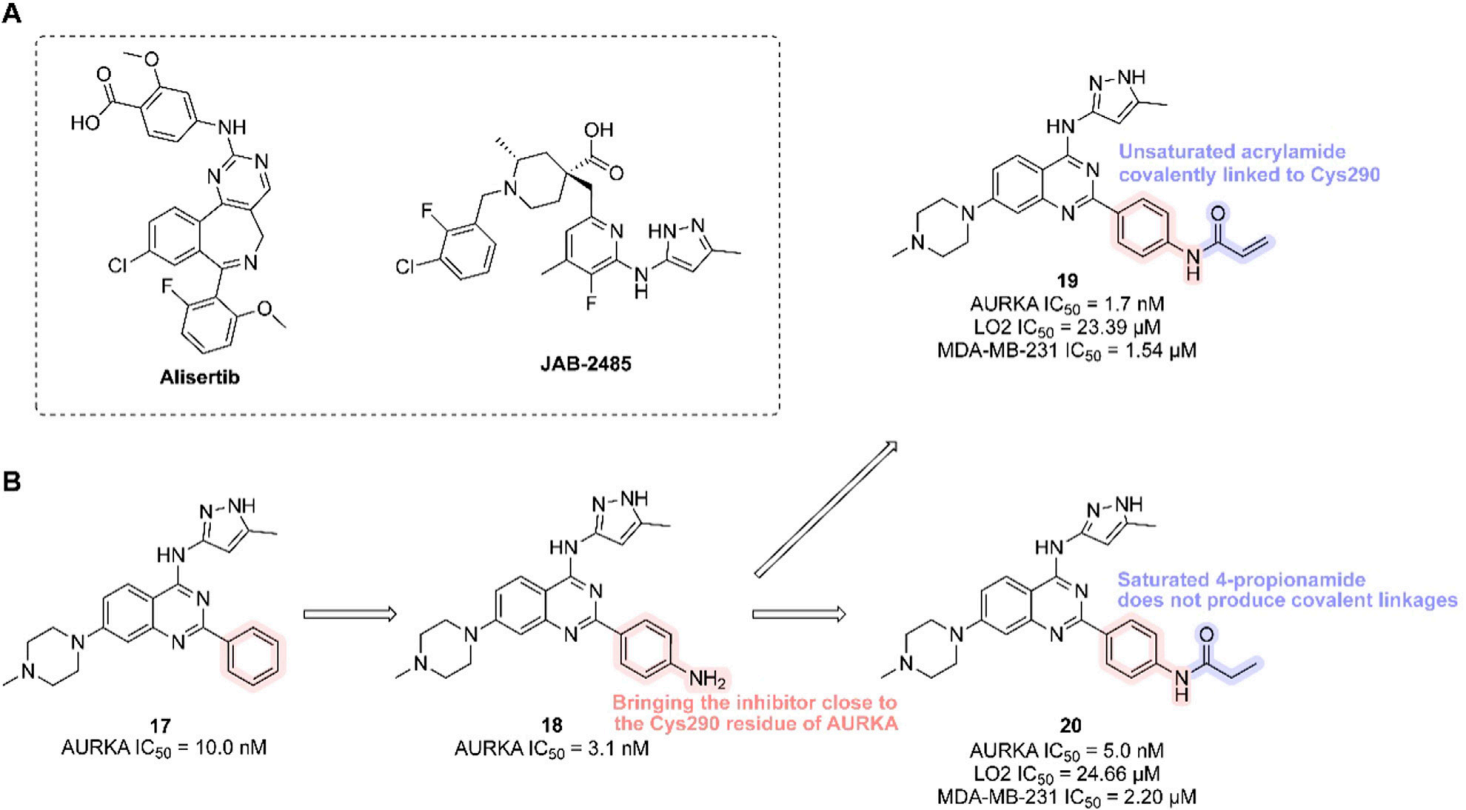
Chemical structures of Alisertib, JAB-2485 and inhibitors **17**-**20**. **(A)** Chemical structures of Alisertib and JAB-2485. **(B)** Structure optimization process of compound **19**.

Alisertib is a highly potent and selective AURKA inhibitor (IC_50_ = 1.2 nM), demonstrating strong anti-tumor activity in preclinical studies and promising clinical efficacy. JAB-2485, a small-molecule AURKA inhibitor with high selectivity (IC_50_ values: AURKA = 0.327 nM, AURKB = 555 nM, AURKC = 507 nM), significantly reduces AURKA phosphorylation and enhances histone H3 phosphorylation. It exhibits favorable pharmacokinetics and potent anti-tumor activity in preclinical models (Figure 6A).

In 2023, researchers reported the discovery of a first-in-class covalent AURKA inhibitor (Zhang et al., 2023). It was found that coenzyme A irreversibly binds AURKA at cysteine residue Cys290. Inspired by this, scientists introduced an–NH_2_ group to the benzene ring of a potent AURKA inhibitor (compound 17, AURKA IC_50_ = 10.0 nM) to position it near Cys290. They then added a covalent warhead—such as acrylamide, acrylate, or sulfuryl fluoride—to form a thioether bond with Cys290, generating a covalent interaction. The resulting compound (compound 19, containing an acrylamide moiety) exhibited the highest *in vitro* activity, selectively inhibiting both normal and cancer cells (LO2 IC_50_ = 23.39 μM; MDA-MB-231 IC_50_ = 1.54 μM), and showed strong inhibition of both AURKA and AURKB (AURKA IC_50_ = 1.7 nM). In contrast, its non-covalent analogue (compound 20) showed lower potency (AURKA IC_50_ = 5.0 nM; MDA-MB-231 IC_50_ = 2.20 μM). Multiple studies confirmed that compound 19 forms a covalent bond with AURKA, validating Cys290-mediated inhibition (Figure 6B). Compound 19 effectively reduced AURKA phosphorylation at Thr288, induced G2/M phase arrest, triggered apoptosis, and suppressed cancer cell migration and proliferation. In an MDA-MB-231 xenograft mouse model, compound 19 demonstrated significant anti-tumor efficacy [15 mg/kg every 2 days, TGI = 30.9%].

#### 3.1.5 ATR/CHK1/WEE1 inhibitors

The Ataxia telangiectasia and Rad3-related (ATR)–checkpoint kinase 1 (CHK1)–WEE1 signaling pathway forms a critical DNA damage response (DDR) mechanism responsible for repairing single-strand breaks (SSBs) and functions as a key component of the G2/M cell cycle checkpoint. Upon activation by various genotoxic stresses (Pilié et al., 2019), ATR is recruited to sites of DNA damage (Cheng et al., 2022), where it phosphorylates and activates CHK1 (Karnitz and Zou, 2015). Subsequently, WEE1 activation and inhibition of the CDC25C phosphatase suppress the cyclin B–CDK1 complex required for mitotic entry, thereby arresting the cell cycle at the G2/M phase to facilitate DNA repair (Jin et al., 2021a; Visconti et al., 2016). In rapidly proliferating tumor cells, alterations or deficiencies in DDR pathways are common, promoting tumorigenesis and uncontrolled proliferation. This may cause tumor cells to become dependent on alternative DDR pathways for survival (Ivy et al., 2016). Therefore, inhibition of key nodes in the ATR–CHK1–WEE1 axis can disrupt DDR, abolish G2/M checkpoint control, induce DNA repair deficiencies in fast-dividing tumor cells, and ultimately trigger cell death. In contrast, normal cells typically retain redundant DDR components that enable them to detect and repair replication errors, rendering them less susceptible to these pharmacological interventions (Cheng et al., 2022).

In summary, the value of ATR/CHK1/WEE1 inhibitors as monotherapies—especially for tumors harboring DDR deficiencies, such as ataxia telangiectasia-mutated gene mutations—has been widely recognized through the concept of synthetic lethality. Importantly, tumors resistant to PARPi often exhibit replication stress or replication-associated DNA abnormalities, resulting in heightened genomic instability (Gaillard et al., 2015). In PARPi-resistant BRCA1-deficient cells, reliance on alternative DDR pathways increases. This vulnerability creates a therapeutic opportunity to target the G2/M checkpoint using ATR/CHK1/WEE1 inhibitors, thereby forcing cells into mitosis despite the presence of DNA damage and overcoming PARPi resistance (Gupta et al., 2022; Kim et al., 2020). Multiple studies have demonstrated that ATR/CHK1/WEE1 inhibitors can enhance the cytotoxic efficacy of PARPi and re-sensitize resistant cell lines to PARP inhibition (Gupta et al., 2022).

##### 3.1.5.1 ATR inhibitors

ATR, a member of the phosphatidylinositol 3-kinase-related kinase (PIKK) family, plays a pivotal role in DNA repair and genome stability maintenance. It functions as a central signaling molecule in the DDR network by orchestrating downstream cell cycle checkpoints and DNA repair events, coordinating multifaceted cellular responses to replication stress (Jin et al., 2021a). However, ATR is frequently overexpressed in breast cancer, and its elevated expression levels are associated with advanced tumor stage and lymphovascular invasion (Abdel-Fatah et al., 2015), underscoring its potential as a therapeutic target. Currently, two ATR inhibitors—Ceralasertib (AZD6738) and Berzosertib (VX-970, M6620)—are under clinical evaluation for breast cancer treatment (Table 3).

**FIGURE 7:**
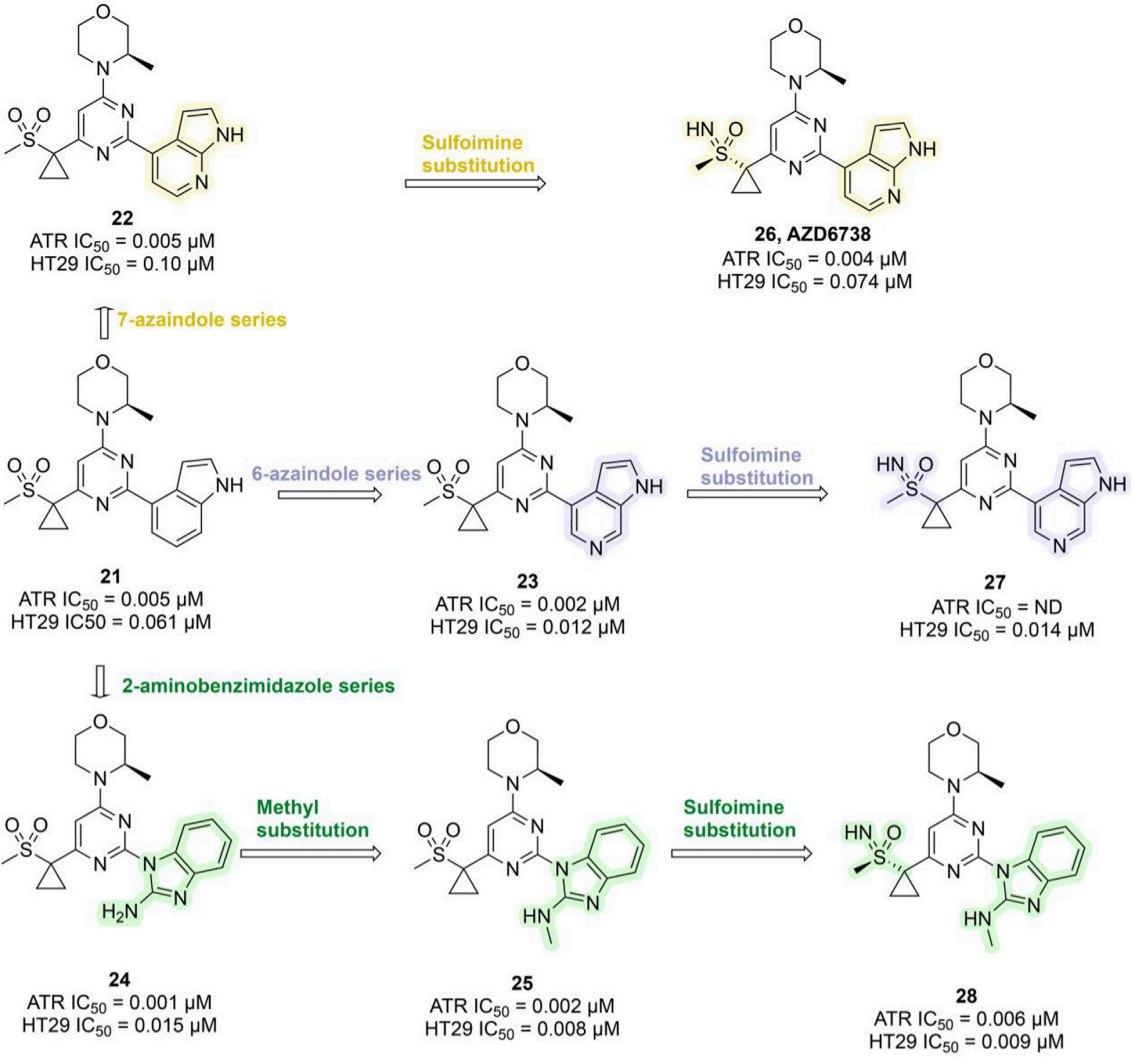
Chemical structures of inhibitors **21–28**.

Ceralasertib, a potent ATR inhibitor, was developed from sulfonylmethyl morpholinopyrimidine compound **21** (ATR IC_50_ = 0.005 μM; HT29 IC_50_ = 0.061 μM). However, its development was initially hampered by poor water solubility and a high risk of drug–drug interactions (DDIs) due to time-dependent inhibition (TDI) of cytochrome P450 3A4 (CYP3A4). Optimization efforts centered on the morpholinopyrimidine pharmacophore, systematically modifying substituents on the pyrimidine ring. This led to the development of the 7-azaindole series (compound **22**, ATR IC_50_ = 0.005 μM), the 6-azaindole series (compound **23**, ATR IC_50_ = 0.002 μM), and the 2-aminobenzimidazole series (compound **24**, ATR IC_50_ = 0.001 μM). In 3-(R)-methylmorpholine-2-aminobenzimidazole 24, replacing the amino group with a methyl group yielded compound **25**, which exhibited enhanced ATR inhibition (ATR IC_50_ = 0.002 μM; HT29 IC_50_ = 0.008 μM), though water solubility remained suboptimal. Researchers subsequently explored sulfoimine substitutions, producing compounds **26–28** (Figure 7). Among them, compound **26**, derived from the 7-azaindole series, stood out with strong ATR inhibitory potency (ATR IC_50_ = 0.004 μM; HT29 IC_50_ = 0.074 μM), excellent selectivity, markedly improved aqueous solubility, and the elimination of CYP3A4 TDI (Foote et al., 2018). Compound **26** demonstrated favorable pharmacokinetic (PK) properties in humans and showed robust tumor growth inhibition in xenograft mouse models. It was named AZD6738 (Ceralasertib) and displayed remarkable antitumor activity both as a monotherapy and in combination with PARPi in preclinical studies (Wilson et al., 2022). AZD6738 has now progressed to Phase III development and is currently being investigated in a Phase II clinical trial (NCT05582538) in combination with durvalumab and nab-paclitaxel for the treatment of advanced TNBC (Table 3).

**FIGURE 8:**
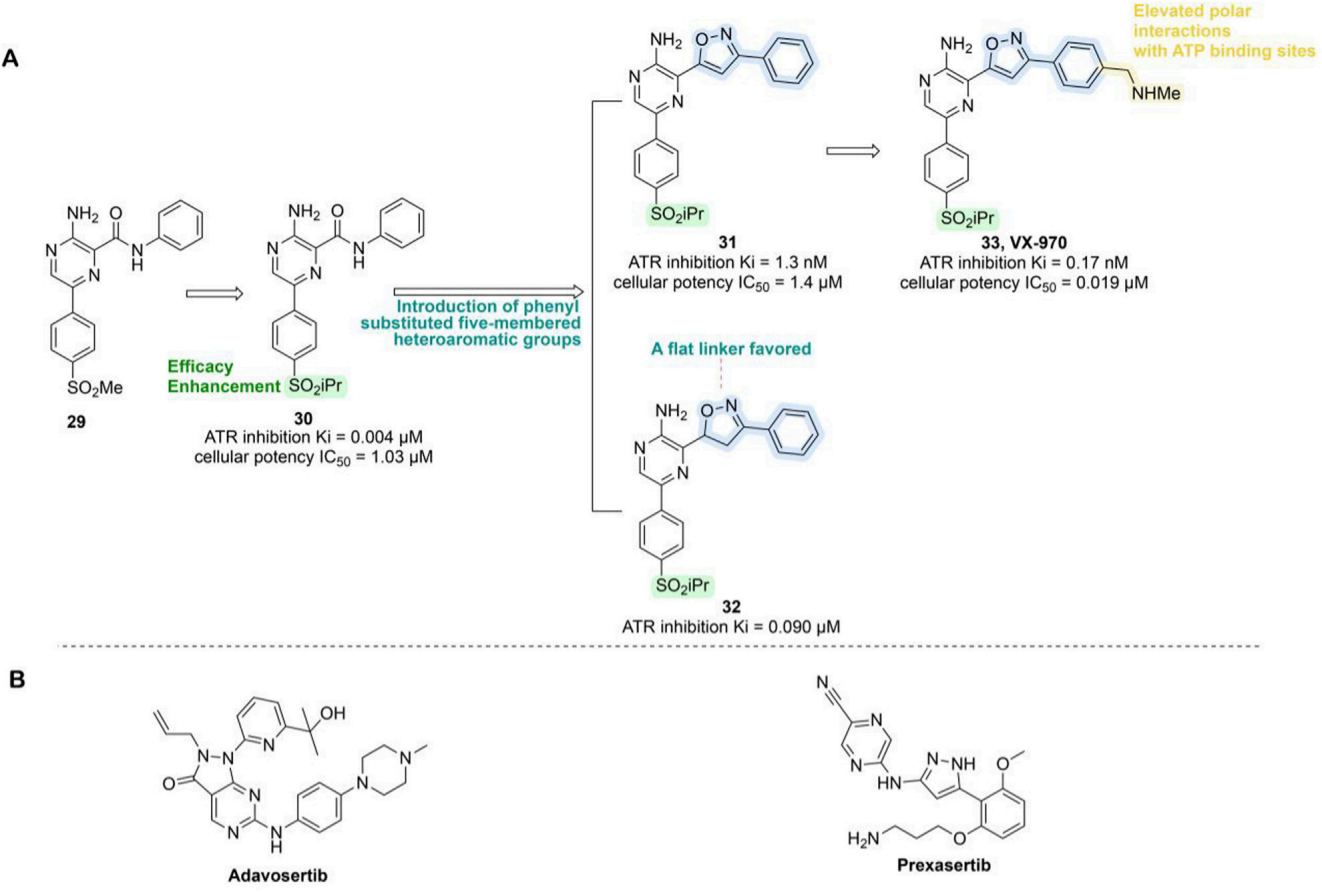
Chemical structures of inhibitors **29**-**33**, Adavosertib and Prexzsertib. **(A)** Structure optimization process of compound **33**. **(B)** Chemical structures of Adavosertib and Prexzsertib.

VX-970 originated from compound **29**, which was based on a 2-aminopyrazine amide hinge binding motif but was limited by suboptimal physical properties and PK characteristics. Optimization efforts primarily targeted modifications of the anilide group, introducing phenyl-substituted heteroaromatic groups to enhance ATR binding and avoid spatial clashes with the PIKK family’s tyrosine gatekeeper residue. Replacing the methylsulfone with isopropylsulfone improved potency (compound **30**, ATR Ki = 0.004 μM, IC_50_ = 1.03 μM). Further refinement revealed that an unsaturated planar isoxazole linker (compound **31**, Ki = 1.3 nM, IC_50_ = 1.4 μM) significantly outperformed the partially saturated analog, confirming the necessity of planar geometry for optimal ATP pocket complementarity. Subsequent incorporation of an N-methylbenzylamine group at the phenylisoxazole para-position (compound **33**, Ki = 0.17 nM, IC_50_ = 0.019 μM) markedly enhanced binding affinity, cellular potency, solubility, and PK profile, sensitizing HCT116 cells to the DNA cross-linking agent cisplatin (Figure 8A) (Knegtel et al., 2019; Gorecki et al., 2020). In pre-clinical models, VX-970 significantly enhances tumor radiosensitivity, particularly in TNBC and metastatic models, where it markedly suppresses tumorigenicity and demonstrates *in vivo* efficacy by inducing tumor regression and prolonging survival (Baschnagel et al., 2021). Clinical investigations of VX-970 have currently advanced to Phase II. In a Phase I clinical trial (NCT04052555), VX-970 in combination with radiotherapy aims to overcome chemotherapy resistance in TNBC or ER+/HER2-breast cancer (Table 3). In another phase I study (NCT02157792), the combination of VX-970 with cisplatin in patients with advanced TNBC and HR+/HER2– breast cancer achieved an objective response rate of 23.4% in the TNBC cohort, including a complete response rate of 4.3% and a partial response rate of 19.1%, with a disease control rate as high as 61.7% (Sofianidi et al., 2024).

##### 3.1.5.2 CHK1 inhibitors

The inhibitory phosphorylation of CDC25A by activated CHK1, followed by its proteasomal degradation, leads to decreased CDK2 activity during the S phase (Qiu et al., 2018). Simultaneously, CHK1 mediates the phosphorylation of CDC25B and CDC25C, promoting their degradation and subsequently inactivating the cyclin B–CDK1 complex (Peng et al., 1997), thereby inducing G2/M phase arrest. However, CHK1 amplification has been observed in multiple cancer types, including breast cancer, and is strongly associated with resistance to chemotherapy or radiotherapy, increased recurrence rates, and poor clinical outcomes. These characteristics highlight CHK1 inhibitors as a promising therapeutic strategy for breast cancer treatment (Neizer-Ashun and Bhattacharya, 2021).

Currently, the CHK1/2-selective inhibitor prexasertib is being evaluated in combination with samotolisib for the treatment of metastatic TNBC (NCT04032080, Table 3). Prexasertib induces HRD in TNBC cells, demonstrating anti-tumor efficacy and synergistic effects when combined with the PARPi olaparib (Mani et al., 2019). Furthermore, the combination of prexasertib with the PI3K/mTOR inhibitor samotolisib has shown synergistic or additive effects in most tumor models, effectively suppressing primary tumor growth in the MDA-MB-231 model and exhibiting preliminary clinical activity in heavily pretreated patients—indicating encouraging therapeutic potential (Hong et al., 2021).

##### 3.1.5.3 WEE1 inhibitors

WEE1 is a tyrosine kinase crucial for regulating the G_2_/M checkpoint by phosphorylating CDK1 to prevent the entry of unrepaired damage into mitosis. It can also modulate replication initiation through CDK2 phosphorylation (Aarts et al., 2012). WEE1 is overexpressed in various cancer types, including breast cancer, leukemia, and melanoma, and may be associated with poor prognosis (Matheson et al., 2016). Studies indicate that inhibiting WEE1 causes tumor cells to prematurely enter mitosis with unrepaired DNA, leading to cell death. Furthermore, anti-estrogen therapies induce cell cycle arrest at the G_1_ phase, and subsets of breast cancer cells resistant to CDK4/6i exhibit defects in the G_1_ checkpoint, relying more on the G_2_/S checkpoint for DNA repair. Thus, inhibiting WEE1 may overcome resistance to standard therapy in ER + breast cancer (Fallah et al., 2021), and it holds significant implications for Rb-deficient, ER + resistant breast cancer cells (Kumarasamy et al., 2022). Bedsides, in BRCA1/2 wild-type TNBC models, combined inhibition of WEE1 and PARP (Olaparib) markedly suppresses tumor growth and reshapes the tumor microenvironment. This regimen induces synergistic anti-proliferative effects across multiple BRCA1/2 wild-type cell lines, characterized by elevated γH2AX levels, increased replication stress, and robust activation of the STING pathway, along with upregulation of IFNβ, CXCL10, and MHC-I, indicating an immune-stimulatory response. In immunocompetent mouse models, the combination not only delays tumor progression and improves survival beyond either monotherapy but also enhances lymphocytes infiltration and overcomes resistance associated with olaparib insensitivity and high WEE1 expression (Teo et al., 2023).

The WEE1 inhibitor Adavosertib (AZD1775, MK1775) was developed through a high-throughput screening program and inhibits WEE1 kinase in an ATP-competitive manner (IC_50_ = 5.2 nM) (Figure 8B). It suppresses the phosphorylation of its downstream substrate CDC2 at Tyr15, thereby abolishing the G2/S checkpoint and synergistically inducing apoptosis in p53-defective tumors or TNBC cells when combined with chemotherapeutic agents, ultimately enhancing anticancer efficacy (Hirai et al., 2009). Additionally, AZD1775 has demonstrated potential to overcome resistance to breast cancer therapies such as trastuzumab (Sand et al., 2020) and CDK4/6i (Fallah et al., 2021), making it an attractive candidate among small-molecule drugs. Currently, AZD1775 is being evaluated in combination with olaparib for the treatment of metastatic TNBC (NCT03330847, Table 3).

### 3.2 Targeting BCSCs related proteins

BCSCs play a critical role in breast cancer metastasis and recurrence due to their capabilities for self-renewal, proliferation, and multilineage differentiation. These properties endow them with enhanced antioxidative capacity, tumorsphere formation, tumorigenicity, chemoresistance, and the ability to contribute to tumor heterogeneity (Song and Farzaneh, 2021; Al-Hajj and Clarke, 2004). Several signaling pathways, including Wnt/β-catenin, JAK/STAT3, and Notch, are dysregulated in BCSCs, leading to the activation of epithelial-to-mesenchymal transition (EMT), tumorigenesis, proliferation, migration, and drug resistance (Xu et al., 2020; Si et al., 2015). Inhibitors targeting these signaling pathways may suppress BCSC activity and thereby help resolve issues related to tumor resistance.

#### 3.2.1 JAK-STAT3 pathway inhibitors

When growth factors, cytokines, or oncogenic proteins such as IL-6 and EGF bind to their homologous receptors on the cell surface, upstream kinases of STAT3, such as JAKs, become activated (Lo et al., 2005). These kinases phosphorylate both the receptors and the cytoplasmic tail of STAT3. Phosphorylated STAT3 then dimerizes and translocates to the nucleus, where it forms complexes with co-activators and binds to promoter regions of target genes, driving their transcription. This process is critical for regulating cell proliferation, differentiation, and apoptosis, as well as for modulating immune cell profiles (Ma et al., 2020; Hashemi et al., 2019), highlighting the importance of studying the key components of this pathway—JAK and STAT3.

##### 3.2.1.1 JAK inhibitors

**FIGURE 9:**
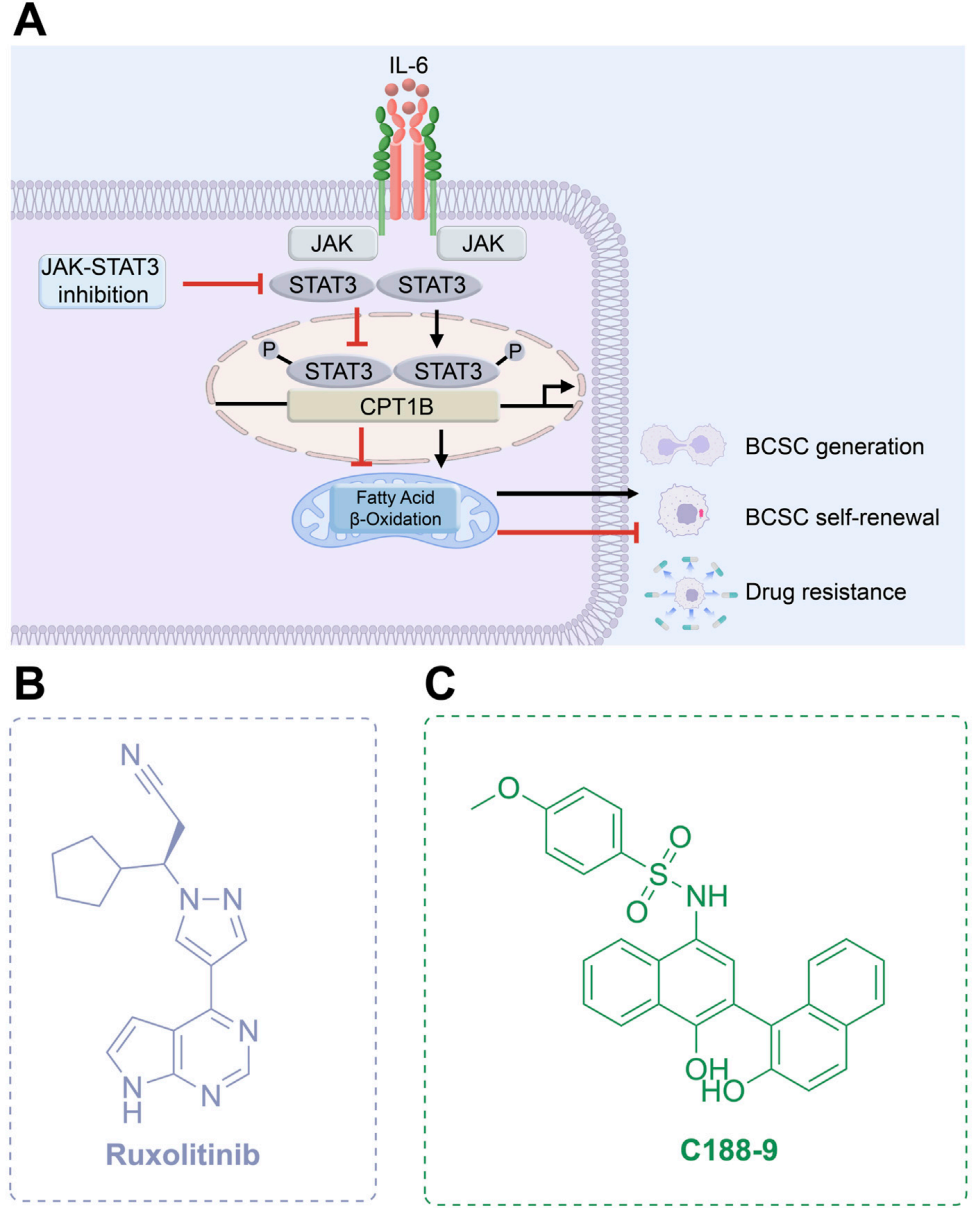
Targeting JAK-STAT3 pathway. **(A)** Mechanism of action of JAK-STAT3 inhibitors. **(B,C)** Chemical structures of Ruxolitinib and C188-9.

JAKs play a central role in activating STAT proteins in breast cancer, acting as key mediators in signal transduction (Quintás-Cardama and Verstovsek, 2013). Additionally, the oncogenic role of JAK2 kinase’s is implicated through alterations in the epigenome (Dawson et al., 2009). Inhibiting JAKs is expected to disrupt STAT function, potentially blocking crosstalk between tumor cells and surrounding cells in the tumor microenvironment, thereby exerting anti-tumor effects. JAK2 inhibitors can also reverse BCSC-mediated drug resistance by directly binding to and inhibiting the P-glycoprotein (P-gp) efflux pump, thereby blocking drug efflux (Long et al., 2024). Thus, targeting JAK2 signaling disrupts stem cell survival pathways and further suppresses efflux activity, enhancing therapeutic efficacy. The JAK1/2 inhibitor ruxolitinib (Figure 9), previously approved for treating diseases like myelofibrosis, is currently being combined with chemotherapy for treating triple-negative inflammatory breast cancer patients (NCT02876302) (Lynce et al., 2024), showing promising progress (Table 3).

##### 3.2.1.2 STAT3 inhibitors

STAT, a transcription factor involved in regulating cellular proliferation and differentiation, is considered one of the most compelling anti-tumor targets, with STAT3 being particularly prominent (Wong et al., 2022). Persistent activation and overexpression of STAT3 have been consistently observed in various cancers, including breast cancer, and are strongly correlated with poor clinical prognosis (Qin et al., 2019). By dysregulating downstream targets involved in cell proliferation, angiogenesis, and other critical biological processes, hyperactivated STAT3 contributes to the initiation and progression of breast cancer (Ma et al., 2020; Valeta-Magara et al., 2019). Furthermore, signaling pathways mediated by hyperactivated STAT3 can promote the formation, stemness, and self-renewal of BCSCs by enhancing fatty acid oxidation, while also driving uncontrolled cell proliferation and survival, thereby fostering chemoresistance in cancer cells (Dong et al., 2021; Wang et al., 2018).

STAT3 can drive resistance to targeted therapies in breast cancer through alternative pathways. Tamoxifen impacts the binding of cyclin D1 with ER and STAT3 in cyclin D1-overexpressing breast cancer cells, abolishing cyclin D1-mediated inhibition of STAT3 and growth suppression, allowing STAT3 to exhibit anti-apoptotic activity. Additionally, activation of ER leads to Tamoxifen resistance (Ishii et al., 2008). Mechanisms underlying endocrine therapy resistance in ER + breast cancer include long non-coding RNAs (lncRNAs) Lnc-DC (Peng et al., 2021), crosstalk with conditional macrophages (Castellaro et al., 2019), upregulation of IL-6 and IL-6R by interfering with the inhibitory activity of NCOR2 (Tsoi et al., 2021), HOXB13 transcriptionally upregulating IL-6 (Shah et al., 2013), or loss of HDAC ZIP (Zhu et al., 2020), which mediates STAT3 activation or signaling enhancement, upregulates anti-apoptotic signals, or activates alternative survival pathways. Additionally, upregulation of the IL-6/STAT3 pathway has been detected in ER + breast cancer resistant to CDK4/6i palbociclib, which mediates the upregulation of EMT and BCSCs pathways. This alteration, along with downregulation of DNA repair pathways and ER, promotes cancer cell resistance (Kettner et al., 2019). Additionally, the anti-apoptotic signals stimulated by STAT3 contribute to the limited response of breast cancer cells to HDAC inhibitors and the HER2 inhibitor lapatinib (Zeng et al., 2016). Persistent activation of STAT3 confers intrinsic resistance to chemotherapy, endocrine therapy, and targeted agents by upregulating survival and metabolic pathways such as MDR1 and fatty acid β-oxidation (e.g., CPT1B/FAO) (Jiang et al., 2024). Moreover, STAT3 activation promotes an immunosuppressive tumor microenvironment by enhancing the infiltration of myeloid-derived suppressor cells and M2 macrophages, inhibiting dendritic cell maturation, and recruiting Tregs. It also stimulates the secretion of pro-tumorigenic cytokines such as IL-6 and VEGF, facilitating angiogenesis and microenvironmental remodeling (Bournazou and Bromberg, 2013). In multiple tumor models, STAT3 inhibition reduces immunosuppressive myeloid populations, increases effector T cell and dendritic cell infiltration, and enhances responsiveness to immune checkpoint blockade (Jin et al., 2021b). Hence, inhibiting the STAT3 signaling pathway may reverse resistance in breast cancer, and offer a novel strategy for managing resistant tumors (Kettner et al., 2019; Huang et al., 2018).

C188-9 (TTI-101) is currently undergoing clinical evaluation (Table 3) for the treatment of metastatic HR+/HER2-breast cancer in patients who have developed resistance to first-line palbociclib plus AI therapy (NCT05384119). C188-9 was developed through a structure-based drug design program that involved docking approximately 920,000 compounds from a computer-generated database into the peptide-binding pocket of the STAT3 SH2 domain. Through virtual ligand screening and subsequent optimization, C188-9 was identified via structural modification as an effective inhibitor of the interaction between STAT3 and pY-peptide (Figure 9) (Kettner et al., 2019; Xu et al., 2009).

#### 3.2.2 Notch signaling pathway inhibitors

**FIGURE 10:**
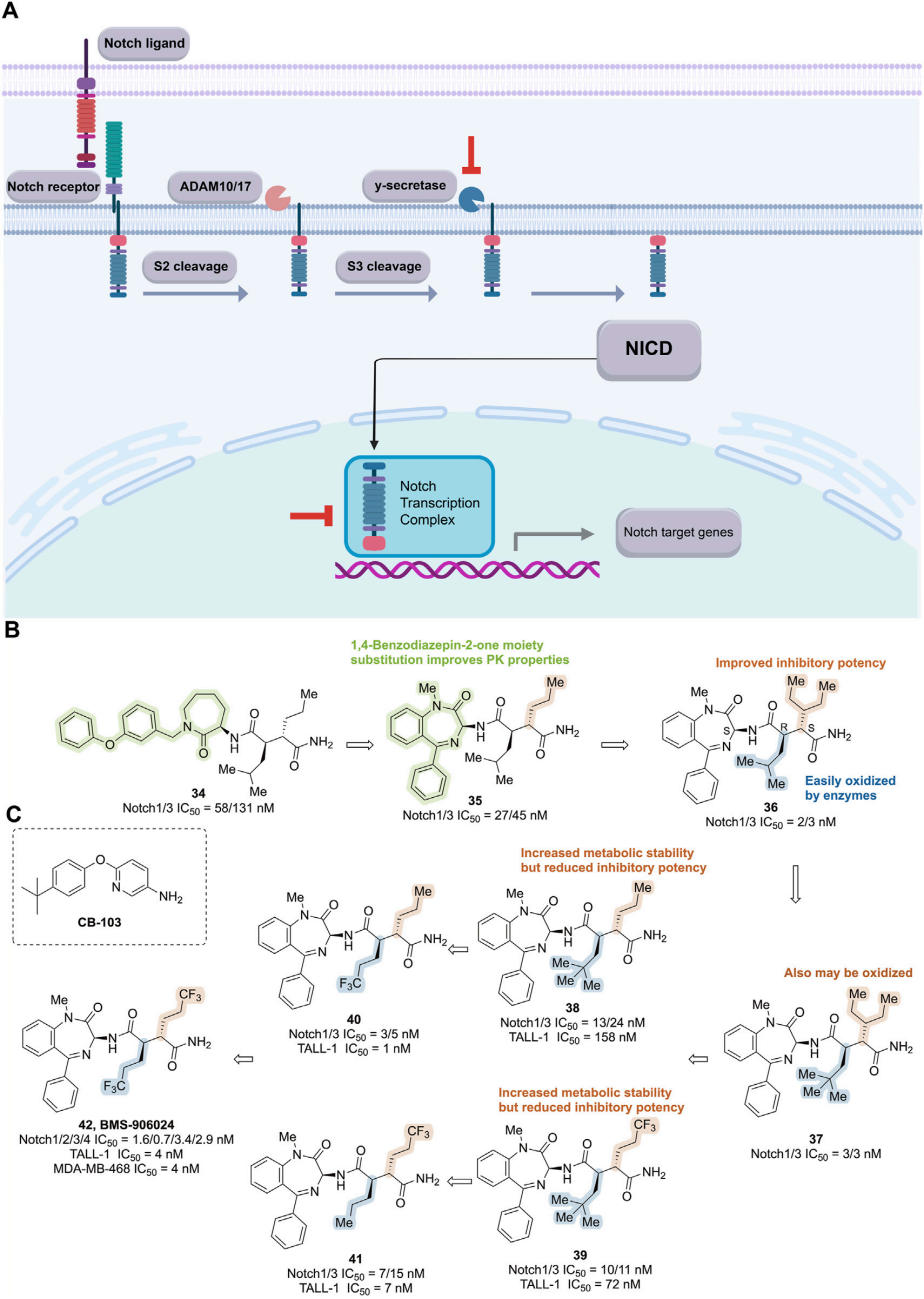
Targeting Notch signaling pathway. **(A)** Mechanism of action of notch pathway inhibitors. **(B)** Structure optimization process of compound **42**. **(C)** Chemical structures of CB-103.

The Notch signaling pathway is essential for maintaining tissue homeostasis during both embryonic development and adulthood. It regulates self-renewal, stem cell quiescence, cell proliferation, apoptosis, activation of differentiation programs, and specific cell fate determination of embryonic and adult stem cells (Krishna et al., 2019). Upon binding of Notch receptor precursors to homologous ligands, a series of proteolytic cleavages are triggered, including the extracellular domain release mediated by amyloid precursor protein (ADAM) and two consecutive cleavages by γ-secretase within the transmembrane domain. These events release the Notch intracellular domain (NICD), which then translocates to the nucleus, binds to DNA-binding proteins, and recruits coactivators to initiate the expression of downstream target genes (Figure 10A) (Kumar et al., 2016; Fortini, 2002).

Overexpression of Notch receptors and ligands has been detected in various human cancers, implicating their involvement in tumor development, angiogenesis, metastasis, recurrence, and drug resistance—all closely associated with poor prognosis (Lamy et al., 2017). Alterations in the Notch pathway typically occur early in disease progression and may significantly contribute to tumor initiation, proliferation, and progression, while correlating with more aggressive and drug-resistant phenotypes (Jia et al., 2021). Furthermore, compelling evidence suggests that the Notch signaling pathway plays a critical role in the maintenance, survival, and self-renewal of BCSCs (Sharma et al., 2015).

The Notch pathway is frequently activated in breast cancer, mediating stress responses and serving as a potential target to overcome multidrug resistance. In endocrine therapy-resistant breast cancer, Notch signaling is dysregulated, promoting resistance through mechanisms such as regulation of ER expression, interaction with other resistance pathways, modulation of tumor macrophage differentiation, increase in the BCSC-to-mesenchymal cell ratio, alteration of the tumor microenvironment, and influence on the cell cycle (Bai et al., 2020; BeLow and Osipo, 2020). Continuous assessment of the Notch-EGFR/HER2 signaling networks reveals that HER-driven breast cancer cells exploit the Notch pathway to sustain HER2-induced signaling via the PI3K pathway, enabling survival against HER2-targeted therapy (Baker et al., 2014). Notch activation also contributes to resistance to PI3K inhibitors (Muellner et al., 2011; Fu et al., 2019), PARPi (Decker et al., 2017), and chemotherapy/radiotherapy. Importantly, BCSCs partially account for treatment resistance. The Notch signal uniquely enriches BCSCs, maintaining their survival and self-renewal, and promoting resistance to targeted or cytotoxic therapies (BeLow and Osipo, 2020).

##### 3.2.2.1 γ-Secretase inhibitors (GSIs)

γ-Secretase activates Notch by cleaving peptide bonds within an intramembrane domain of the Notch protein, allowing the cleaved NICD to translocate into the nucleus. The intramembrane activity of γ-secretase also releases other biologically relevant membrane proteins associated with normal or pathological processes (McElroy et al., 2007). GSIs act as pan-Notch suppressors by inhibiting NICD production and Notch target gene transcription, thereby reducing activated Notch levels and its substrate proteins within cells (Wen et al., 2022). Currently, the monotherapy GSI AL101 (BMS-906024, osugacestat) is used to treat patients with recurrent or metastatic TNBC characterized by Notch activation (NCT04714619), aiming to evaluate the drug’s efficacy and safety (see Table 3).

During the development of BMS-906024, caprolactam **34**, a potent inhibitor of Notch-1 and -3 receptors, was identified through high-throughput cell-based transcriptional reporter assays (Notch1/3 IC_50_ = 58/131 nM). Substitution of caprolactam with a 1,4-benzodiazepin-2-one moiety resulted in compound **35** with improved pharmacokinetic properties, while further optimization led to compound **36**, which exhibited compromised metabolic stability (Notch1/3 IC_50_ = 2/3 nM, TALL-1 IC_50_ = 3 nM). Biotransformation studies revealed that the methine functionality on the isobutyl group was most susceptible to enzymatic oxidation. However, adding methyl groups to the isobutyl moiety of **36** (compound **37**) did not enhance oxidative stability, indicating that the three-pentyl group might also undergo oxidation. Subsequent studies substituted the isobutyl group with propyl (**38**, TALL-1 IC_50_ = 158 nM) or trifluoropropyl (**39**, TALL-1 IC_50_ = 72 nM), improving metabolic stability but reducing inhibitory potency against cell proliferation. Further optimization by altering the alkyl group on the succinamide in 38 led to compound 40, which demonstrated enhanced potency while maintaining metabolic stability (Notch1/3 IC_50_ = 3/5 nM, TALL-1 IC_50_ = 1 nM). Modification of 39 yielded the regioisomeric compound **41**, which showed a promising combination of potency against Notch 1/3 and TALL-1 (Notch1/3 IC_50_ = 7/15 nM, TALL-1 IC_50_ = 7 nM) along with metabolic stability. Combining both modifications resulted in bis(trifluoropropyl) succinimide **42**, which exhibited exceptionally high *in vitro* potency (Notch1/2/3/4 IC_50_ = 1.6/0.7/3.4/2.9 nM, TALL-1 IC_50_ = 4 nM) and complete oxidative stability (Figure 10B). It demonstrated robust anti-tumor activity in Notch-driven leukemia and TNBC xenograft models, earning it the designation BMS-906024 for clinical investigation (Gavai et al., 2015).

##### 3.2.2.2 Notch transcription complex inhibitors

The released NICD translocates into the nucleus, where it combines with DNA-binding proteins and recruits co-activators to form transcriptional complexes. Inhibiting this transcriptional complex impedes Notch pathway activation. The small molecule CB-103, combined with the non-steroidal AIs anastrozole or letrozole (Figure 10C), is being investigated for treating HR+/HER2-advanced breast cancer patients who have previously benefited from AI therapy (NCT04714619, Table 3).

The compound [6-(4-(tert-butyl) phenoxy) pyridine-3-amine], known as CB-103, was identified through high-throughput library screening combined with computer-aided self-organizing mapping programs. CB-103 exhibits favorable absorption, distribution, metabolism, excretion, and PK properties. It selectively disrupts Notch-mediated signaling of all four Notch receptors in a dose-dependent manner by interfering with the assembly of the Notch transcriptional complex. This results in downregulation of Notch transcriptional effector molecules, induction of cell cycle arrest and apoptosis, thereby hindering the proliferation of human tumor cell lines, including GSI-resistant TNBC cell lines such as HCC1187, which harbor chromosomal translocations and Notch gene rearrangements (Lehal et al., 2020).

### 3.3 Targeting Bcl-2 family protein

The B-cell lymphoma-2 (Bcl-2) family of proteins plays a critical role in regulating the intrinsic pathway of cell apoptosis. This family is classified into three categories: multi-domain pro-apoptotic proteins (Bax and Bak); anti-apoptotic proteins (Bcl-2, Bcl-xL, Mcl-1, Bcl-b, and Bcl-w); and pro-apoptotic BH3-only proteins, which are further divided into sensitizers (Bad, Bik, and Noxa) and activators (Bid, Bim, and Puma) (Suvarna et al., 2019).

**FIGURE 11:**
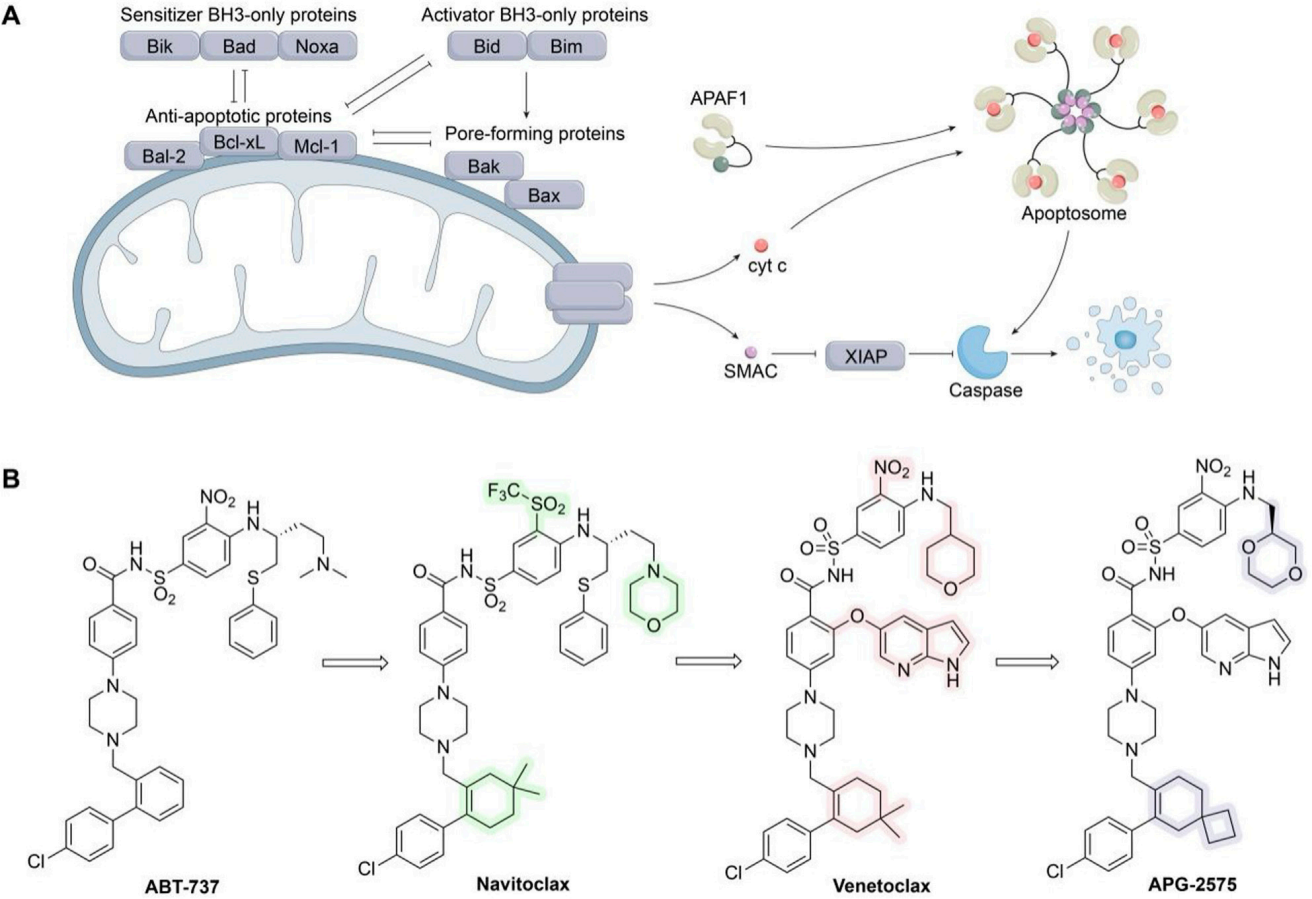
Targeting Bcl-2 family protein. **(A)** Bcl-2 family of proteins and their role in the intrinsic apoptotic pathway. **(B)** Chemical structures of ABT-737, Navitoclax, Venetoclax, and APG-2575.

The intrinsic apoptotic pathway is triggered in response to cellular damage and is activated by most anticancer drugs (Czabotar et al., 2014). It is initiated by various signaling pathways and upregulated following apoptotic signals received by BH3-only proteins. Sensitizer BH3-only proteins bind to anti-apoptotic Bcl-2 members, alleviating their inhibitory effects on Bax and Bak, while activator BH3-only proteins can directly bind to and activate Bax/Bak (Moldoveanu et al., 2014). Activation of Bax and Bak leads to mitochondrial outer membrane permeabilization, releasing second mitochondria-derived activator of caspases (SMAC) and cytochrome c from the intermembrane space into the cytoplasm. Cytochrome c interacts with apoptotic protease-activating factor 1 (APAF1) to form apoptosomes, which bind and activate procaspase-9, subsequently triggering caspase-9 and downstream caspases-3 and -7, ultimately inducing cell apoptosis (Montero and Letai, 2018). Additionally, SMAC released into the cytoplasm inhibits X-linked inhibitor of apoptosis protein (XIAP), which binds caspase-3 and -7 (Figure 11A) (Suvarna et al., 2019).

Resistance to apoptosis is a hallmark of all cancers, including breast cancer (Hanahan and Weinberg Robert, 2011), with dysregulation of the Bcl-2 family playing a pivotal role. Increasing evidence indicates that overexpression of anti-apoptotic Bcl-2 family members, such as Bcl-2, Bcl-xL, and Mcl-1, is a major factor in breast cancer initiation (Adams and Cory, 2018), and is often associated with poor prognosis. These proteins may also synergize with other oncogenes to promote tumor development, metastasis, recurrence, and other malignant characteristics, making them attractive targets for breast cancer therapy (Adams et al., 2018). BH3 mimetics, which mimic the BH3 domain of BH3-only proteins, bind to the hydrophobic groove of anti-apoptotic proteins, inhibit their activity, displace and release pro-apoptotic proteins, thereby inducing cell apoptosis and exerting anti-tumor effects (Zhang et al., 2021).

Furthermore, dysregulation of the anti-apoptotic proteins in Bcl-2 family is implicated in drug resistance in breast cancer. Most anti-tumor drugs induce cellular stress, effectively activating the intrinsic pathway and leading to tumor regression. However, one mechanism by which cancer cells evade pro-apoptotic signals is through the upregulation of anti-apoptotic proteins, thereby constraining pro-apoptotic proteins. This makes the Bcl-2 family a central node for resistance arising from various signaling pathways. Therefore, inhibition of anti-apoptotic proteins with specific inhibitors will downregulate anti-apoptotic signals, not only inhibiting tumor growth but also reversing resistance induced by other drugs (Montero and Letai, 2018; Kawiak and Kostecka, 2022). Upregulation of anti-apoptotic proteins Bcl-2, Bcl-xl, and Mcl-1 in endocrine therapy-resistant (Williams et al., 2018), CDK4/6i-resistant (Whittle et al., 2020), and HER2 TKI-resistant (Eustace et al., 2018) breast cancer cell lines validates this theory. In preclinical studies, inhibiting these proteins has been confirmed to rescue resistant breast cancer or sensitize tumors to respective drugs. Thus, targeting Bcl-2 anti-apoptotic members has emerged as one of the most promising approaches in breast cancer therapy (Table 3). *In vitro* and *in vivo*, the pan-Bcl-2 antagonist simultaneously inhibits Bcl-2, Mcl-1, Bcl-xL, and BFL-1, thereby robustly activating caspase-3/7 and caspase-9 while upregulating pro-apoptotic effectors Bax, PUMA, and Bim—effectively re-engaging apoptosis and partially reversing chemoresistance. Critically, it also eradicates the BCSC subpopulation and suppresses sphere formation by inhibiting the IL-6/STAT3 pathway and downregulating stemness-associated factors (Hu et al., 2018). Currently, the selective Bcl-2 inhibitor venetoclax is used in combination with palbociclib and letrozole to treat ER+/Bcl-2+ locally advanced or metastatic breast cancer patients (NCT03900884). APG-2575 is currently being investigated in clinical trials either as a monotherapy for advanced solid tumors or in combination with the CDK4/6i palbociclib for ER+/HER2-metastatic breast tumors that have progressed or relapsed following first-line treatment (NCT04946864). TQB3909 is undergoing clinical evaluation in patients with HR+/HER2-advanced breast cancer (NCT05775575). The Bcl-2/Bcl-xl inhibitor navitoclax is also used in combination with olaparib for TNBC patients with somatic or germline mutations in BRCA1/2 (NCT05358639).

Navitoclax (ABT-263) has been strategically modified at three key positions of ABT-737, achieving subnanomolar affinity for inhibiting Bcl-2, Bcl-xL, and Bcl-w. These modifications result in a 20-fold improvement in the PK/pharmacodynamic relationship between oral exposure and *in vitro* efficacy in human tumor cell lines (FL5.12 Bcl-2 EC_50_ = 5.9 nM, FL5.12 Bcl-xL EC_50_ = 4.2 nM), leading to complete tumor regression in xenograft models (Park et al., 2008). However, on-target thrombocytopenia resulting from Bcl-xL inhibition limits the clinical efficacy of this drug. To address this, venetoclax (ABT-199) was developed by removing the thiophenyl unit and introducing an azaindole group via an ether linkage into the central core of ABT-263. Venetoclax is a highly potent, orally bioavailable, and Bcl-2-selective inhibitor (FL5.12 Bcl-2 EC_50_ = 4 nM, FL5.12 Bcl-xL EC_50_ = 261 nM) (Souers et al., 2013). Nevertheless, many patients treated with venetoclax experience hematological toxicity and tumor lysis syndrome. APG-2575, a novel BH3 mimetic Bcl-2-selective inhibitor, was developed by replacing the dimethyl group of venetoclax with a cyclobutyl ring and substituting the tetrahydro-2H-pyran group with 1,4-dioxane. APG-2575 rapidly penetrates tumor cells and disrupts the Bcl-2–Bim complex, ultimately inducing Bax/Bak-dependent, caspase-mediated apoptosis, and demonstrating potent antitumor activity (Deng et al., 2022).

In addition to the inhibitors mentioned above, the development of Mcl-1 inhibitors has gradually emerged as a research focus (Wang et al., 2021). Moreover, researchers are exploring the therapeutic application of activating pro-apoptotic proteins in cancer treatment, thereby reinvigorating the therapeutic potential of the Bcl-2 family.

### 3.4 Other novel agents

#### 3.4.1 CDK9 inhibitors

CDK9 plays a pivotal role in regulating transcriptional elongation, while its impact on the cell cycle is minimal. CDK9 associates with cyclin T to form the positive transcription elongation factor b (P-TEFb), thereby activating its kinase activity. It phosphorylates the CTD of RNA Polymerase II, DRB sensitivity-inducing factor (DSIF), and negative elongation factor (NELF), facilitating transcription essential for the efficient expression of most genes (Egloff, 2021). CDK9 activity is significantly upregulated in various cancers, enhancing the overexpression of downstream regulatory genes and promoting tumor progression (Mandal et al., 2021).

**FIGURE 12:**
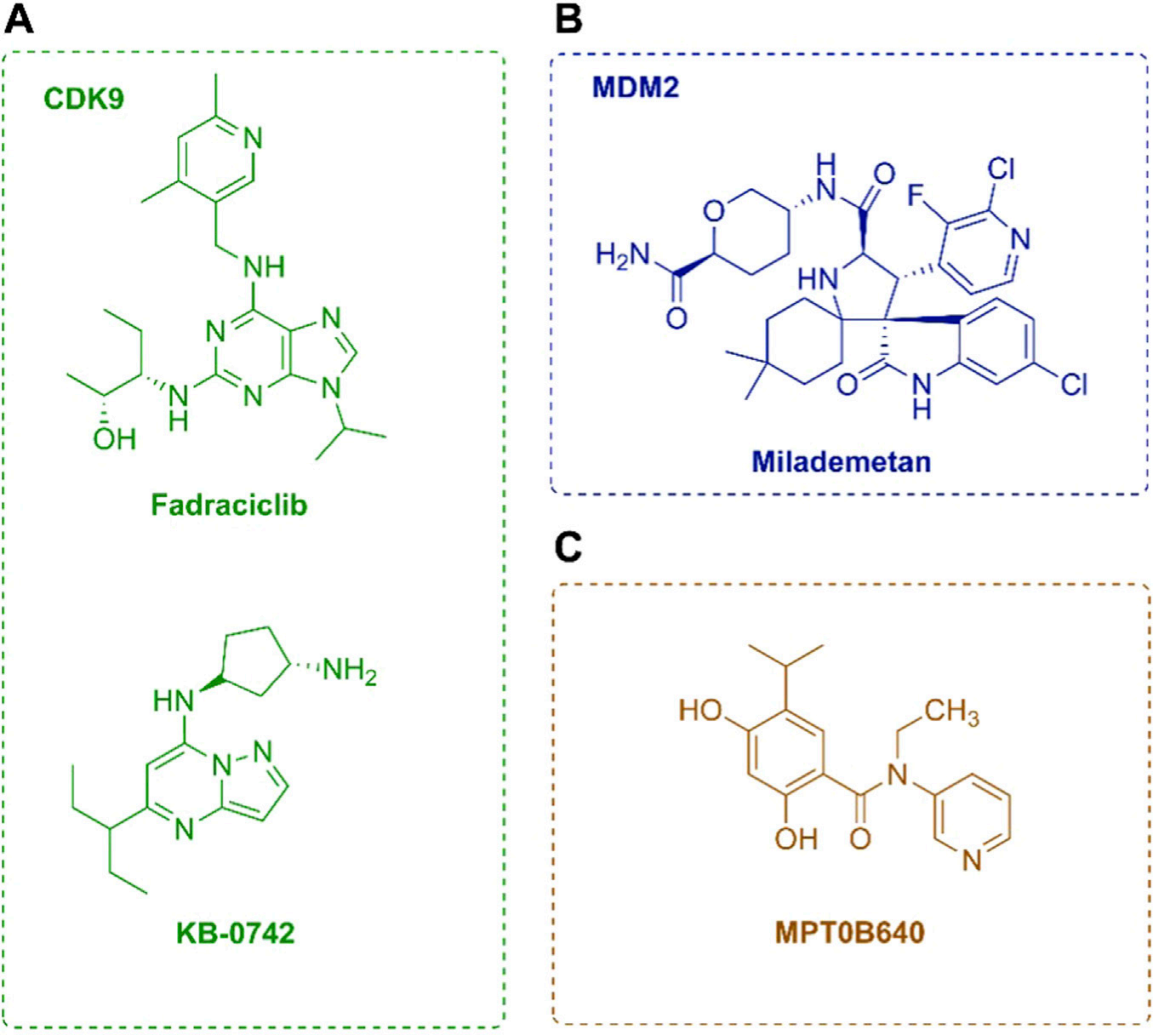
Chemical structures of several novel agents. **(A)** Chemical structures of Fadraciclib and KB-0742. **(B)** Chemical structures of Milademetan. **(C)** Chemical structure of MPT0B640.

Investigations into CDK9’s role in promoting drug resistance in breast cancer are rapidly expanding. In endocrine-resistant ERα+ breast cancer, CDK9 mediates hyperphosphorylation of serine-2 in the RNA Pol II CTD at the c-MYC gene promoter, resulting in transcriptional elongation and overexpression of c-MYC, which drives estrogen-independent growth of resistant cancer cells (Sengupta et al., 2014). Furthermore, increased CDK9 mRNA expression in plasma-derived exosomes from HR+/HER2-breast cancer patients treated with palbociclib plus hormonal therapy correlates with clinical resistance (Del et al., 2019). Research also suggests that anaplastic lymphoma kinase (ALK) directly phosphorylates CDK9 at tyrosine-19, enhancing its kinase activity and nuclear localization. This extends P-TEFb function and activates polymerase II-dependent transcription of homologous recombination repair genes, contributing to PARPi resistance (Chu et al., 2022). Consequently, CDK9 inhibition sensitizes breast cancer to endocrine therapy, CDK4/6i, and PARPi. Currently, the safety and pharmacology of the CDK2/9 dual-target inhibitor Fadraciclib (NCT04983810), as well as selective CDK9 inhibitors KB-0742 (NCT04718675) and PRT2527 (NCT05159518), are being evaluated in patients with solid tumors, including breast cancer (Table 3; Figure 12A). Notably, Fadraciclib’s clinical evaluation includes HR+/HER2− metastatic breast cancer patients who have previously been treated with CDK4/6i.

#### 3.4.2 SHP2 inhibitors

Src homology region 2-containing protein tyrosine phosphatase 2 (SHP2) plays a positive regulatory role in signal transduction triggered by growth factors and cytokines. As a central node for multiple signaling pathways, SHP2 modulates diverse cellular events, including cell proliferation and migration (Asmamaw et al., 2022; Zhang et al., 2015). SHP2 contributes significantly to tumorigenesis in several cancers, including breast cancer, where it participates in cancer cell initiation, invasion, metastasis, and drug resistance, making it an attractive therapeutic target (Song et al., 2021).

SHP2 has been linked to resistance to targeted therapies in breast cancer. First, it inhibits the MAPK pathway, helping overcome FGFR-induced resistance to endocrine therapy and CDK4/6i (Mao et al., 2020). Second, increased SHP2 phosphorylation is observed in breast cancer cells resistant to the HER2 TKI neratinib, where it acts as a shared signaling node that integrates inputs from the extracellular matrix and receptor tyrosine kinases (RTKs), thereby promoting drug resistance and metastasis (Chen et al., 2020). Another study suggests that activation of the SHP2/GAB1-MEK axis enables HER2+ breast cancer cells to evade inhibition by lapatinib (Ding et al., 2020). Furthermore, SHP2 overexpression induces resistance to PI3K inhibition and is frequently activated during resistance development to PI3K inhibitors. Therefore, targeting SHP2 can disrupt RTK- and MAPK-mediated drug resistance pathways in breast cancer (Heynen et al., 2022). Currently, the selective SHP2 inhibitor JAB-3312 is being assessed in two breast cancer-related clinical trials (NCT04121286, NCT04045496), indicating promising development potential (Table 3).

#### 3.4.3 RAD51 inhibitors

RAD51 is a core protein in the repair of DSBs through homologous recombination, a process vital for preserving genomic integrity. The strand invasion and exchange by RAD51, facilitated by BRCA2, which coats single-stranded DNA to initiate pairing with double-stranded DNA, represent a crucial step in homologous recombination (Demeyer et al., 2021). Additionally, as a DNA-binding protein, RAD51 regulates nucleases, helicases, DNA translocases, and signaling proteins to modulate replication stress responses, such as replication fork reversal (Bhat et al., 2018). RAD51 is frequently overexpressed in breast cancer and plays key roles in tumor cell development and survival. Its dysregulation may contribute to genomic instability in cancer, making RAD51 inhibition a promising therapeutic strategy (Wang et al., 2022).

Restoring homologous recombination in breast cancer cells and stabilizing replication forks are key pathways that lead to resistance to PARPi. Multiple studies indicate that the upregulation of the core protein RAD51 is crucial in this process through various mechanisms. Directly targeting RAD51 to inhibit its activity is a key strategy for enhancing cancer cell sensitivity to PARPi and overcome resistance (Clements et al., 2018; Moustafa et al., 2021). Moreover, combined inhibition of RAD51 and PARP can expand the therapeutic window of PARPi (Liu et al., 2017; Zhao et al., 2017). In ER + breast cancer, methylation of BRCA2 partially inhibits RAD51, resulting in increased RAD51 expression and resistance to AIs (Jia et al., 2019). Therefore, developing RAD51 inhibitors is significantly important. *In vitro* and *in vivo* studies demonstrate that in TNBC cells, RAD51 inhibitors reduce the IC_50_ of cisplatin by approximately fourfold. In xenograft models, cisplatin monotherapy inhibits tumor growth by only 33%, whereas its combination with RAD51 inhibitors reduces tumor volume by over 50%, indicating strong synergy and a marked reversal of resistance. Additionally, novel RAD51 inhibitors such as OA-NO_2_ alkylate critical residues on RAD51, disrupting its binding to ssDNA and impairing homologous recombination repair (Tsang and Munster, 2022). Currently, a first-in-class RAD51 inhibitor, CYT-0851, is undergoing a Phase I/II clinical trial (NCT03997968), recruiting patients with refractory B-cell malignancies and solid tumors, including breast cancer (Table 3).

#### 3.4.4 Polθ inhibitors

Polθ, encoded by POLQ, plays a pivotal role in microhomology-mediated end joining (MMEJ), facilitating the repair of DSBs (Gourley et al., 2019). Elevated expression of Polθ has been observed in 70% of breast cancers and several other cancer types, where it promotes error-prone repair and may contribute to cancer progression (Chen and Pomerantz, 2021). Notably, MMEJ functions as a compensatory repair mechanism in cancers with homologous recombination repair deficiencies, such as those harboring BRCA1/2 mutations. This results in an increased dependence of cancer cells on Polθ activity, rendering Polθ a promising therapeutic target for the treatment of homologous recombination-deficient cancers (Pismataro et al., 2023).

Dysregulated expression of Polθ may lead to resistance to PARPi and chemotherapy (Lavudi et al., 2023). BRCA-deficient cancer cells have been shown to rely more on Polθ expression for survival in the presence of genotoxic agents and PARPi. Patients resistant to PARPi due to BRCA1 mutations exhibit an MMEJ signature, suggesting that Polθ may drive this resistance. Combined inhibition of PARP and Polθ leads to synergistic anti-tumor effects and potentially delays the development of PARPi resistance (Ceccaldi et al., 2015). Specific Polθ inhibitors, such as the antibiotic novobiocin and ART558, demonstrate potent cytotoxicity against PARP-resistant homologous recombination-deficient tumors and enhance the efficacy of PARPi (Zhou et al., 2021; Zatreanu et al., 2021). Therefore, Polθ inhibitors may represent a strategy to overcome cancer resistance to PARPi. Preclinical studies demonstrate that the next-generation Polθ inhibitor ART558 (IC_50_ = 7.9 nM) induces extensive DNA damage and synthetic lethality in BRCA1/2-deficient tumor cells, while also synergizing with PARPi like olaparib. Importantly, ART558 retains potent activity in models with PARPi resistance due to 53BP1/Shieldin complex loss—by inhibiting the TMEJ pathway, it drives ssDNA accumulation and DNA damage even in resistant backgrounds, effectively restoring tumor sensitivity (Zatreanu et al., 2021). Currently, the selective Polθ inhibitor ART6043 is being evaluated in a Phase I/II clinical trial (NCT05898399), recruiting patients with HER2-negative advanced breast cancer harboring BRCA mutations (Table 3). In one arm of another study (NCT04991480), HER2-negative breast cancer patients will receive treatment with ART4215, while another arm of the study recruited patients who have previously undergone PARPi therapy (Table 3).

#### 3.4.5 MDM2 inhibitors

Mouse double minute 2 (MDM2) is an E3 ubiquitin ligase that interacts with the major tumor suppressor and pro-apoptotic factor p53. It inhibits the transcriptional activity of p53 by targeting it for proteasomal degradation, thereby preventing cell cycle arrest or apoptosis (Haupt et al., 2017). Elevated levels of MDM2, which suppress p53 function, are frequently observed in cancers, including ER + breast cancer, and serve as an independent prognostic marker in breast cancer (Turbin et al., 2006). Dysregulated MDM2 promotes breast cancer invasion and metastasis through p53-independent pathways. Thus, antagonizing the p53-MDM2 interaction by inhibiting MDM2 represents a promising therapeutic strategy for inducing apoptosis in tumors (Wang et al., 2017).

In a clinical investigation, patients treated with CDK4/6i in combination with endocrine therapy demonstrated increased amplification of MDM2 gene copies. This suggests that MDM2 inhibitors may be a crucial strategy to overcome resistance to endocrine therapy and CDK4/6i in ER + breast cancer (Rao et al., 2023). Preclinical studies have confirmed that MDM2 inhibitors activate wild-type p53 in fulvestrant-resistant breast cancer models, effectively inducing p21 expression and cell cycle arrest, thereby promoting apoptosis and inhibiting tumor growth (Lu et al., 2016). Although the combination of MDM2 inhibitors with endocrine therapy or CDK4/6i does not significantly enhance apoptosis, it profoundly modulates cell cycle-related transcriptional programs. This exerts synergistic effects on resistant cell lines by increasing antagonism toward cell cycle progression (Portman et al., 2020). Additionally, it has been found that MDM2, in conjunction with the ubiquitin E3 ligase HUWE1, plays a broader and p53-independent role in regulating cellular apoptosis. It suppresses apoptosis in lapatinib-resistant HER2+ breast cancer, indicating a potential role for MDM2 inhibition in overcoming lapatinib resistance (Kurokawa et al., 2013). Studies have also revealed that inhibiting MDM2 expression reduces DNA repair mediated by homologous recombination and sensitizes breast cancer cells to PARPi olaparib (Herok et al., 2021). Larger-scale compound screening further reveals strong synergy between MDM2 inhibition and blockade of the MAPK (MEK) or PI3K pathways. Additionally, co-treatment with Bcl-2 or HDAC inhibitors significantly enhances antitumor efficacy (Saiki et al., 2014). These findings highlight the broad potential of MDM2-based combination strategies to overcome therapeutic resistance and inform rational design of clinical regimens. Currently, the MDM2 inhibitor milademetan is being combined with fulvestrant to treat ER+/HER2-advanced breast cancer patients with GTAG3 mutations in tumors and/or ctDNA (Figure 12B). These patients have progressed after prior therapies (including CDK4/6i) (NCT05932667, Table 3).

#### 3.4.6 Heat shock protein 90 (Hsp90) inhibitors

Hsp90 is one of the most abundant intracellular chaperone proteins (Lu et al., 2012). It relies on its intrinsic ability to bind and hydrolyze ATP—facilitated by cochaperones—to mediate the folding and maturation of over 300 client proteins, including EGFR, HER2, Akt, CDK4/6, and ER, through a conformationally ATPase-driven cycle. Hsp90 is essential for maintaining the stability and activity of these client proteins, playing a critical role in cellular signaling networks (Li L. et al., 2020; Butler et al., 2015; Ali et al., 2006). Under stress conditions, Hsp90 expression is markedly upregulated, promoting aberrant folding and overexpression of client proteins, which contributes to tumorigenesis (Li L. et al., 2020). Moreover, Hsp90 is overexpressed in many cancers, including breast cancer, and is associated with tumor malignancy and drug resistance (Butler et al., 2015). Inhibition of Hsp90 disrupts the Hsp90–client protein complex, resulting in a rapid decline and degradation of client protein activity. This ultimately leads to the simultaneous depletion of multiple oncogenic proteins in breast tumors and the combinatorial downregulation of signals propagated through diverse oncogenic pathways (Sumi and Ghosh, 2022).

Hsp90 inhibitors can simultaneously degrade multiple oncoproteins and inhibit multiple signaling pathways, making them highly promising in overcoming tumor resistance, including in breast cancer. When existing therapies reduce reliance on the originally targeted pathway due to target mutations or alternative survival pathways, the abundant client proteins of Hsp90 often participate in regulating these new pathways. Hsp90 inhibitors degrade client proteins, disrupting the reliance of tumors on branched signaling and addressing downstream target resistance. For instance, all oncogenic tyrosine kinases, including their native forms, drug-resistant mutations, and other activated forms, are clients of Hsp90. This characteristic enables Hsp90 inhibitors to effectively overcome breast cancer resistance caused by secondary mutations of HER2 (Wang et al., 2016). Numerous clients of Hsp90, including survivin (Brady et al., 2015) and proteins of alternative HER2 survival pathways (Ye et al., 2021), also contribute to reversing lapatinib resistance in HER2-positive breast cancer cells. Similarly, the degradation of client proteins Akt and HER2 can reverse resistance to endocrine therapy (Bai et al., 2017). Additionally, disrupting key DNA repair proteins, such as BRCA1 (including its mutants), BRCA2, and RAD51, can restore the sensitivity of resistant cells to PARPi (Johnson et al., 2013; Jiang et al., 2017). In HER2-positive breast cancer, the Hsp90 inhibitor 17-AAG (tanespimycin) combined with trastuzumab has shown the ability to effectively reverse therapeutic resistance (Lu et al., 2012). In a phase II clinical trial involving 31 patients with trastuzumab-refractory disease, the combination achieved a 22% objective response rate and a 59% clinical benefit rate (stable disease plus partial response) (Modi et al., 2011). Leveraging the multi-client nature of Hsp90, this combinatorial approach broadens the therapeutic window and offers a strategy to overcome resistance associated with single-target therapies. Currently, the Hsp90 inhibitor MPT0B640 (JIN-001) is being evaluated for its safety and anti-tumor activity in patients with late-stage solid tumor, including those with breast cancer (NCT04294875, Table 3) (Figure 12C).

#### 3.4.7 KAT6 inhibitors

KAT6 proteins are members of the MYST acetyltransferase family (Huang et al., 2016), which regulate chromatin organization by modifying lysine residues on histones and play a crucial role in gene expression regulation (Voss and Thomas, 2018). KAT6A and KAT6B interact with bromodomain-PHD finger proteins (BRPF1/2/3) through their MYST domains (Ullah et al., 2008). They subsequently mediate interactions with inhibitor of growth family member 5 (ING5) (or its homolog ING4) and MYST/Esa1-associated factor 6 (MEAF6), forming complexes that acetylate lysine residues on histone H3 tails (Yan et al., 2020). Aberrant histone acetylation can contribute to tumorigenesis and cancer progression; thus, dysregulated expression of KAT6 proteins promotes tumor development (Trisciuoglio et al., 2018). A metagenomic analysis of 37 lysine acetyltransferases across various human cancer types, with a focus on breast cancer, revealed that KAT6A is frequently subject to genomic amplification or mutation (Jiang et al., 2020).

The amplification/overexpression rate of KAT6A/6B in breast cancer has been reported to reach 10%–15%. These proteins can directly regulate the expression level of Erα and contribute to the progression of ER+/HER2-breast cancer (Yu et al., 2017). Overexpression of KAT6A is associated with poor clinical prognosis in ER+/HER2-breast cancer patients (Turner-Ivey et al., 2014). Inhibitors of KAT6A can block ERα expression at the transcriptional level, potentially overcoming endocrine therapy resistance caused by ESR1 mutations. Additionally, combining these inhibitors with CDK4/6i or new oral SERDs is expected to enhance therapeutic efficacy. In a preclinical trial, the KAT6A/B inhibitor reduces RNA Pol II binding, which is associated with antitumor activity, and downregulates genes involved in estrogen signaling, cell cycle, Myc, and stem cell pathways. Moreover, these inhibitors demonstrated significant TGI in ER + breast cancer *in vivo* models, including those resistant to endocrine therapy (Sharma et al., 2023). Clinical investigations have showed that combining KAT6A/B inhibitors with Fulvestrant was effective regardless of the mutation status of ESR1 and PIK3CA/PTEN/Akt1 gene. Furthermore, this combined treatment significantly reduced the variant allele fraction level of ESR1, suggesting its overall effectiveness (Mukohara et al., 2024). Recent clinical data indicate that KAT6A/B inhibitors exhibit promising activity in advanced ER-positive breast cancer. As monotherapy, they achieve disease stabilization in a subset of patients; when combined with Fulvestrant, the objective response rate reaches 30%, with a median progression-free survival of 10.7 months (Mukohara et al., 2024). The KAT6A/B inhibitor PF-07248144 is currently being validated for safety, tolerability, and clinical efficacy in combination with either Fulvestrant, letrozole + palbociclib, or PF-07220060 + Fulvestrant in patients with advanced or metastatic solid tumors, including breast cancer (NCT04606446, Table 3).

Although targeted therapies are designed to act on specific molecular pathways, tumor cells frequently develop resistance through convergent mechanisms. Common resistance features include enhanced DDR capacity, upregulation of anti-apoptotic signaling (e.g., overexpression of Bcl-2 family proteins), maintenance of cancer stem cell phenotypes, and increased activity of drug efflux pumps (Safa, 2020). These mechanisms often converge on shared molecular nodes such as cell cycle and DNA damage response kinases (e.g., CDK2/7, ATR/CHK1, WEE1), anti-apoptotic proteins (e.g., Bcl-2, Bcl-xL), and stemness-associated survival pathways (e.g., JAK–STAT3, Notch), extending to oncogenic transcription factors like Myc (Li and Dong, 2024). To address these challenges, several cross-pathway resistance-overcoming strategies have emerged. These include exploiting synthetic lethality—for example, targeting BRCA-deficient tumors with PARPi (Tiek and Cheng, 2022); dual inhibition of compensatory signaling pathways, such as co-targeting hormone receptors and cell cycle regulators (Shieh, 2022); and activating anti-tumor immunity—for instance, PARP inhibition can trigger the cGAS/STING pathway and upregulate PD-L1 expression, thereby enhancing CD8^+^ T-cell infiltration. Such mechanistic convergence and therapeutic strategies provide a conceptual framework for rational combination therapies aimed at overcoming resistance across drug classes.

## 4 Conclusions and future perspectives

In recent years, the rapid expansion of clinically available breast cancer drugs has ushered in a new era of treatment. Targeted small molecule inhibitors, recognized for their efficacy and structural flexibility, have been approved for clinical use and have significantly improved patient survival and prognosis (Barchiesi et al., 2020). However, resistance to existing therapeutic agents limits their long-term effectiveness, a challenge further complicated by the complexity of resistance mechanisms and the heterogeneity of breast cancer (Zhou et al., 2023). Therefore, elucidating the mechanisms underlying drug resistance in breast cancer and developing novel therapeutic strategies to overcome it are of critical importance.

This review examines the resistance mechanisms associated with clinically available targeted small molecules in breast cancer. In essence, targeting shared signaling nodes involved in biological activity holds promise for overcoming drug resistance in breast cancer (Bai et al., 2020; Chen et al., 2020; Attia et al., 2020). Future research will prioritize understanding these resistance mechanisms and identifying convergent pathway nodes to devise focused strategies for combating drug-resistant breast cancer. Potential therapeutic targets for addressing drug-resistant breast cancer can be classified according to upstream inputs and downstream signaling pathways, including cell cycle checkpoint components (CDK2, CDK7, Mps1, AURKA, ATR, CHK1, and WEE1); BCSC-related molecules (JAK, STAT3, and proteins in the Notch pathway); and apoptosis-related proteins (Bcl-2, Bcl-w, and Bcl-xl). Small molecule inhibitors targeting CDK2, CDK7, Mps1, AURKA, STAT3, Bcl-2, CDK9, and MDM2 show strong potential in overcoming endocrine therapy or CDK4/6i resistance and are currently under clinical investigation. For TNBC, which has the poorest prognosis, emerging therapies targeting JAK, γ-secretase, ATR, CHK1, WEE1, RAD51, and Polθ offer new therapeutic options for patients with limited treatment alternatives (Qin et al., 2019). Notably, many of these therapeutic targets are also implicated in resistance to chemotherapy and radiotherapy (Li S. et al., 2020; Ji et al., 2019), extending the potential impact of these treatments beyond specific targeted therapies.

Despite promising preclinical and early clinical results, the clinical development of anti-resistance drugs remains in its early stages (Table 4). Consequently, the pathway toward applying these agents in patients who have failed first-line treatments remains long and complex. Moreover, the targets and related drugs discussed in this review are primarily focused on overcoming endocrine therapy and CDK4/6i resistance in HR + breast cancer. Further research is required to identify additional therapeutic targets that can benefit patients with HER2+ and TNBC subtypes. In parallel, balancing the efficacy and safety of small molecule inhibitors poses a major challenge. Several inhibitors have been withdrawn from clinical trials due to significant toxicity, which continues to be a substantial barrier to successful clinical translation (Said et al., 2022). A key strategy to address this issue lies in enhancing drug selectivity (Freeman-Cook KD. et al., 2021)—designing molecules that specifically target essential receptors without interfering with similar proteins. This optimization is largely driven by structural modifications of small molecules, guided by high-resolution 3D protein structures and computational docking tools. By leveraging differences in molecular shape, electrostatics, conformation, and flexibility—and by employing covalent, non-competitive, and allosteric binding strategies based on the binding modes between molecules and target proteins—off-target effects can be rationally minimized. In addition to traditional approaches such as structure-based drug design, high-throughput screening, and virtual screening, novel therapeutic modalities such as proteolysis-targeting chimeras (PROTACs) and molecular glues (Guo et al., 2024), as well as innovative screening platforms like DNA-encoded compound libraries, are increasingly shaping the landscape of drug discovery. Furthermore, with the growing maturity of artificial intelligence and gene editing technologies, their integration into drug development is poised to more effectively guide the design and optimization of next-generation inhibitors (Li and Song, 2020).

**TABLE 4:** Summary of emerging therapeutic agents for reversing drug resistance in breast cancer: Mechanisms of action and limitations.

Drug class	Target	Clinical stages	Resistance-reversal mechanism	Limitation
CDK2 inhibitor	CDK2	I, II	It can overcome resistance to endocrine therapy or CDK4/6i, primarily by inducing cellular senescence to suppress tumor proliferation. For instance, resistance can be reversed through RBL2 dephosphorylation and C-MYC–driven senescence, effectively eliminating CDK4/6-resistant clones	No CDK2 inhibitor has been approved to date, largely due to its high homology with CDK1, which hampers selectivity and leads to significant toxicities such as myelosuppression (Zeng et al., 2024); the absence of precise biomarkers and compensatory upregulation of other cyclins (e.g., Cyclin E) contribute to resistance development
CDK7 inhibitor	CDK7	I, II	As a transcriptional kinase for RNA polymerase II, sustains the expression of multiple resistance-associated kinases and proliferative genes. In HER2-resistant breast cancer models, CDK7 inhibitors disrupt HER2/PI3K/AKT-driven kinase reprogramming, synergize with HER2 inhibitors, and effectively reverse resistance	Its inhibitors often exhibit broad off-target effects, dose-limiting toxicity, and inconsistent efficacy, leading to the termination of some clinical trials due to safety concerns (Gong and Li, 2024); resistance may also arise through the activation of compensatory transcriptional pathways
Mps1 inhibitor	Mps1	I, II	Mps1 inhibition induces chromosomal instability and mitotic catastrophe, promoting lethal genomic missegregation. By enhancing multipolar spindle formation and chromosome misalignment, Mps1 inhibitors effectively eliminate drug-resistant tumor cells	The narrow therapeutic window of Mps1 inhibitors—due to the close proximity of effective and toxic doses—limits their monotherapy potential and necessitates combination with agents like CDK4/6 inhibitors to spare normal cells (Martinez et al., 2015); certain tumors may acquire tolerance to aneuploidy by suppressing checkpoint mechanisms, such as APC/C inactivation
AURKA inhibitor	AURKA	I, II	AURKA governs mitosis and cancer cell self-renewal; its inhibition disrupts spindle formation in resistant cells and eradicates stem-like populations. In taxane-resistant TNBC models, targeting AURKA suppresses stemness markers such as ALDH1, thereby restoring chemosensitivity	Early AURKA inhibitors showed limited monotherapy efficacy and substantial toxicity, such as lethargy and decreased neutrophils (Dees et al., 2011); at tolerable doses, objective response rates were low, with most patients achieving only disease stabilization
ATR/CHK1/WEE1 inhibitor	ATR/CHK1/WEE1	I, II	Inhibiting DNA damage checkpoints forces highly replicative tumor cells into mitosis, triggering catastrophic DNA damage; under HR-deficient conditions, this induces synthetic lethality and enhances sensitivity to PARPi	These agents exhibit notable toxicity, primarily myelosuppression (neutropenia/thrombocytopenia) and gastrointestinal side effects (Gorecki et al., 2021); their efficacy is tightly linked to DDR status, necessitating predictive biomarkers; tumors may evade cytotoxicity via alternative DNA repair pathways
JAK-STAT3 pathway inhibitor	JAK, STAT3	I, II	This pathway drives the expression of multiple pro-survival and anti-apoptotic genes. Its inhibition downregulates cytokine signaling and anti-apoptotic proteins such as Bcl-2, thereby reversing drug resistance	Lacking high selectivity, it often triggers broad immune and inflammatory responses; no definitive predictive biomarkers have been identified to date
Notch signaling pathway inhibitor	γ-secretase, Notch Transcription Complex	I, II	Involved in maintaining breast cancer stem cells and promoting resistance-related EMT, Notch signaling inhibition eradicates CSCs and reverses EMT, thereby restoring sensitivity to endocrine and chemotherapeutic agents	Induce severe gastrointestinal (e.g., goblet cell hyperplasia) and dermatologic toxicity, resulting in a narrow therapeutic window; as monotherapies, they exhibit limited tumor response and typically require combination strategies; a lack of highly selective agents targeting individual Notch receptors remains a major limitation
Targeting Bcl-2 family protein	Bcl-2	I, II	It can dismantle anti-apoptotic barriers, restoring apoptotic sensitivity in resistant cancer cells	Monotherapy shows limited efficacy, as tumors often evade apoptosis by upregulating bypass proteins such as MCL-1 and Bcl-xL, necessitating combination strategies; hematologic toxicity remains the primary safety concern (Martin and Dowsett, 2013)
CDK9 inhibitor	CDK9	I, II	CDK9, a key transcriptional elongation kinase, promotes resistance by sustaining short-lived oncogenic transcripts. Its inhibition rapidly downregulates labile RNAs such as MYC and MCL-1, thereby inducing apoptosis in resistant cells	CDK9 is essential for normal transcription, and its inhibition carries risks of myelosuppression and gastrointestinal toxicity; current inhibitors lack sufficient selectivity, showing differential effects primarily under high replicative stress or in combination regimens (Mustafa et al., 2024); development remains in early stages, with long-term resistance mechanisms yet to be elucidated
SHP2 inhibitor	SHP2	I	SHP2 inhibition disrupts feedback activation induced by receptor tyrosine kinases (e.g., HER2, FGFR) and the extracellular matrix, thereby preventing reactivation of resistance-driving signaling pathways	Most SHP2 inhibitors are allosteric and show limited efficacy in tumors harboring SHP2 mutations; systemic inhibition may impair normal tissue function, particularly in the immune system; oral bioavailability and pharmacokinetic properties remain suboptimal and require further refinement
RAD51 inhibitor	RAD51	I, II	It disrupts homologous recombination repair and, when combined with PARPi, mimics BRCA deficiency in HR-proficient tumors to induce synthetic lethality	Clinically relevant RAD51 inhibitors remain in early development, with most compounds exhibiting limited potency; as normal cells also rely on RAD51 for DNA repair, sustained inhibition may increase genomic instability in healthy tissues; advancing this strategy requires overcoming challenges of selectivity and toxicity
Polθ inhibitor	Polθ	I, II	Polθ inhibition induces extensive DNA damage and apoptosis in BRCA1/2-mutant tumors; it can also restore synthetic lethality in PARP inhibitor–resistant cells with acquired defects in pathways such as 53BP1 loss	Current inhibitors remain experimental and unapproved; long-term effects are unknown, and tumors may evade lethality by upregulating alternative repair pathways such as canonical NHEJ
MDM2 inhibitor	MDM2	I, II	By reactivating p53, MDM2 inhibition induces p53-dependent cell cycle arrest and apoptosis, offering a strategy to overcome therapeutic resistance	Effective only in tumors with wild-type TP53; ineffective in those with p53 loss or mutation; the primary dose-limiting toxicity is thrombocytopenia, driven by p53-mediated apoptosis of megakaryocytes (Fabbroni et al., 2024)
Hsp90 inhibitor	Hsp90	I	Capable of simultaneously degrading key proteins across multiple pathways—including HER2, EGFR, and Akt—thereby broadly suppressing resistance mechanisms; effectively eliminates both tumor cells and cancer stem-like populations to inhibit drug-resistant tumor growth	Induces a heat shock response with compensatory upregulation of HSP70, attenuating therapeutic efficacy; common toxicities include retinal damage and cardiotoxicity (Reynolds et al., 2025); poor oral bioavailability and unfavorable safety or efficacy profiles have led to the termination of multiple clinical trials
KAT6 inhibitor	KAT6	I, II	KAT6 inhibition downregulates ER target genes, including ESR1 itself, and disrupts the ER transcriptional complex, thereby markedly suppressing the proliferation of endocrine-resistant tumors	Monotherapy shows limited activity, with efficacy primarily observed when combined with endocrine therapy; common adverse effects include hematologic suppression, such as neutropenia (Mukohara et al., 2024)

In this review, we adopt a clinically oriented perspective to explore the potential of novel targeted small molecule inhibitors currently in clinical trials for overcoming drug resistance in breast cancer, with the aim of providing theoretical support for the development and selection of future clinical candidates.

## Glossary

ADAM: amyloid precursor protein
AI: aromatase inhibitor
Akt: protein kinase B
ALK: anaplastic lymphoma kinase
APAF1: apoptotic protease-activating factor 1
AR: aromatase
ATR: ataxia telangiectasia and Rad3-related
ATP: adenosine 5′-phosphate
AURKA: aurora kinase A
Bcl-2: B-cell lymphoma-2
BCSC: breast cancer stem cell
BRCA1/2: breast cancer susceptibility gene 1/2
CAK: cyclin-activating kinase
CDC25A: cell division cycle 25 A
CDK: cyclin-dependent kinase
CDK4/6i: CDK4/6 inhibitors
CHK1: checkpoint kinase 1
CIP/KIP: CDK interaction protein/kinase inhibitor protein
CTD: C-terminal domain
CYP3A4: cytochrome P450 3A4
DDI: drug-drug interaction
DDR: DNA damage repair
DSB: DNA double-strand break
DSIF: DRB sensitivity-inducing factor
EGFR: epidermal growth factor receptor
EMT: epithelial-to-mesenchymal transition
ER: estrogen receptor
EREs: estrogen response elements
GSIs: γ-Secretase inhibitors
HDAC: histone deacetylase
HER2: human epidermal growth factor receptor 2
HR: hormone receptor
HRD: homologous recombination deficiency
HSP90: heat shock protein 90
INK4: inhibitor of CDK4
ING5: inhibitor of growth family member 5
JAK: Janus kinase
LBD: ligand-binding domain
lncRNAs: long non-coding RNAs
MAPK: Mitogen-activated protein kinase
MMD2: mouse double minute 2
MMEJ: microhomology-mediated end-joining
Mps1: monopolar spindle 1
mTOR: mammalian target of rapamycin
mTORC1: mTOR complex 1
mTORC2: mTOR complex 2
NELF: negative elongation factor
NHEJ: non-homologous end-joining
NICD: notch intracellular domain
NAD: nicotinamide adenine dinucleotide
PARP: poly ADP-ribose polymerase
PARPi: PARP inhibitors
PDK1: phosphoinositide-dependent protein kinase 1
P-gp: P-glycoprotein pump
PI3K: phosphatidylinositol 3-kinase
PIK3CA: phosphatidylinositol 3-kinase catalytic subunit
PTEN: phosphatase and tensin homolog
PIKK: phosphatidylinositol 3-kinase-related kinase
PK: pharmacokinetic
PLK1: polo-like kinase one
Polθ: polymerase theta
PROTACs: proteolysis-targeting chimeras
P-TEFb: positive transcription elongation factor b
Rb: retinoblastoma
RNA pol II: RNA polymerase II
SAC: spindle assembly checkpoint
SERDs: selective estrogen receptor degraders
SERMs: selective estrogen receptor modulators
SHP2: src homology containing protein tyrosine phosphatase 2
SMAC: second mitochondria-derived activator of caspases
SSBs: single-strand breaks
STAT3: signal transducers and activators of transcription 3
TDI: time-dependent inhibition
TFIIH: transcription factor IIH
TGI: tumor growth inhibition
TKIs: tyrosine kinase inhibitors
TNBC: triple-negative breast cancer
XIAP: X-linked inhibitor of apoptosis protein
